# Curricula for empathy and compassion training in medical education: A systematic review

**DOI:** 10.1371/journal.pone.0221412

**Published:** 2019-08-22

**Authors:** Sundip Patel, Alexis Pelletier-Bui, Stephanie Smith, Michael B. Roberts, Hope Kilgannon, Stephen Trzeciak, Brian W. Roberts

**Affiliations:** 1 Department of Emergency Medicine, Cooper University Health Care, Cooper Medical School of Rowan University, Camden, NJ, United States of America; 2 Institutional Research and Outcomes Assessment, Philadelphia College of Osteopathic Medicine, Philadelphia, PA, United States of America; 3 Center for Humanism, Cooper Medical School of Rowan University, Camden, NJ, United States of America; 4 Department of Medicine, Cooper University Health Care, Cooper Medical School of Rowan University, Camden, NJ, United States of America; University of Vienna, AUSTRIA

## Abstract

**Background:**

Empathy and compassion are vital components of health care quality; however, physicians frequently miss opportunities for empathy and compassion in patient care. Despite evidence that empathy and compassion training can be effective, the specific behaviors that should be taught remain unclear. We synthesized the biomedical literature on empathy and compassion training in medical education to find the specific curricula components (skills and behaviors) demonstrated to be effective.

**Methods:**

We searched CENTRAL, MEDLINE, EMBASE, and CINAHL using a previously published comprehensive search strategy. We screened reference lists of the articles meeting inclusion criteria to identify additional studies for potential inclusion. Study inclusion criteria were: (1) intervention arm in which subjects underwent an educational curriculum aimed at enhancing empathy and/or compassion; (2) clearly defined control arm in which subjects did not receive the curriculum; (3) curriculum was tested on physicians (or physicians-in-training); and (4) outcome measure assessing the effect of the curriculum on physician empathy and/or compassion. We performed a qualitative analysis to collate and tabulate effects of tested curricula according to recommended methodology from the Cochrane Handbook. We used the Cochrane Collaboration’s tool for assessing risk of bias.

**Results:**

Fifty-two studies (total n = 5,316) met inclusion criteria. Most (75%) studies found that the tested curricula improved physician empathy and/or compassion on at least one outcome measure. We identified the following key behaviors to be effective: (1) sitting (versus standing) during the interview; (2) detecting patients’ non-verbal cues of emotion; (3) recognizing and responding to opportunities for compassion; (4) non-verbal communication of caring (e.g. eye contact); and (5) verbal statements of acknowledgement, validation, and support. These behaviors were found to improve patient perception of physician empathy and/or compassion.

**Conclusion:**

Evidence suggests that training can enhance physician empathy and compassion. Training curricula should incorporate the specific behaviors identified in this report.

## Introduction

Empathy and compassion are foundational elements of the practice of medicine and vital cornerstones of high quality health care.[1, 2] They are closely related terms, with empathy defined as the ability to sense, feel, and understand another's emotions, and compassion defined as an emotional response to another’s pain or suffering involving an authentic desire to help.[3, 4] Both are essential in the care of patients, in that empathy (i.e. understanding of patient suffering) is required to spur compassion (i.e. the emotional response involving action aimed at alleviating patient suffering).[5] As such, in patient care the constructs of empathy and compassion, although distinct, are inextricably linked.

Empathetic and compassionate care has been demonstrated to be associated with improved clinical outcomes for patients.[6–11] For example, empathetic and compassionate care is associated with superior patient adherence to prescribed therapies.[8] In addition, empathetic and compassionate care may reduce depression and improve quality of life.[12–14] Further, among oncology patients a compassionate intervention was found to significantly reduce patient anxiety.[15] Among health care providers, empathetic and compassionate care has been associated with lower burnout and improved well-being.[16] Alternatively, there is a potential emotional cost to identifying too closely with patient distress.[17, 18] Interestingly, functional magnetic resonance imaging (fMRI) studies have found that when a person experiences empathy the pain centers of the brain are activated,[19] whereas when a person focuses on compassion the reward pathways are activated.[19, 20] These data suggest that while experiencing empathy alone may result in negative outcomes for clinicians, integrating compassion training may foster clinician well-being. Within health care systems compassionate care is associated with lower health care costs (e.g. better patient communication resulting in lower spending on unnecessary diagnostic tests and referrals).[21] Despite abundant evidence supporting the importance of compassionate patient care, there is currently evidence to suggest that health care is experiencing a compassion crisis (i.e. an absence of–or inconsistency in–compassionate patient care),[6] in which physicians miss the majority of opportunities to show compassion,[22] instead focusing on narrow biomedical inquiry and explanations.[23]

Empathy and compassion are not simply inherent traits, which health care providers intrinsically either do or do not possess, but can be enhanced through training interventions.[2, 24, 25] Previous studies have demonstrated that empathy and compassion decline during both medical school and residency training,[26–28] with more recent studies now bringing this empathy and compassion decline into question.[29–31] These more recent results may reflect medical curricula starting to focus more on empathy/compassion training, thus attenuating the empathy/compassion decline. However, there is currently no standard for empathy/compassion training and thus there is an urgent need to further develop evidence-based training curricula, which can be implemented during medical training, as well as help inform currently practicing physicians. The first step in developing evidence-based curricula is to identify the specific skills and behaviors that ought to be taught and how best to transfer this knowledge to the learner.

The objectives of this systematic review are to collate the world’s literature on empathy and compassion training in medical education to determine (1) the specific skills and behaviors that should be taught (i.e. have been demonstrated to enhance patient perception of compassion), and (2) the methods of training that are most effective.[32] The results of this report will help inform the development of evidence-based curricula for empathy and compassion training in medical education.

## Methods

### Protocol and registration

We developed and published a systematic review protocol [32] in accordance with the Cochrane Handbook for systematic reviews of interventions,[33] and the Preferred Reporting Items for Systematic Reviews and Meta-Analysis Protocols (PRISMA-P) statement.[34] Our final results are reported according to the PRISMA guidelines.[35] This systematic review was registered in the PROSPERO international prospective register of systematic reviews (registration number CRD42018095040).

### Search for and identification of studies

Our electronic search included databases generally considered to be the most important sources:[33] CENTRAL, MEDLINE, EMBASE, and CINAHL. The search strategies were established using a combination of standardized terms and key words (including empathy, compassion, and derivations thereof), and the fully reproducible search strategy was previously published.[32] We also performed recommended techniques for systematic reviews of complex evidence: we reviewed reference lists of the included articles to identify additional studies for potential inclusion, used electronic citation tracking, and consulted experts in the field.[36] The final search was performed on Feb 1^st^, 2019.

### Eligibility criteria

We included all clinical studies of educational curricula that were described as either empathy training or compassion training. We included both on the grounds of the inter-relatedness and inter-dependence of these constructs as described above, and the fact that training to improve empathy (i.e. the understanding component) typically also improves compassion (i.e. the action component), and training to improve compassion would likely require improving empathy. Further, it would not be possible to perform a rigorous systematic review of one without the other, in that most training programs in this domain involve enhancement of both understanding patients’ emotions and taking action with behaviors toward patients.

As stated in our previously published protocol the inclusion criteria for studies were: (1) an intervention arm in which subjects clearly underwent an educational curriculum aimed at enhancing empathy or compassion; (2) a clearly defined control arm in which subjects did not receive the curriculum (e.g. wait-list, before/after, standard training); (3) the curriculum was tested on physicians, or physicians-in-training; and (4) an outcome measure assessing the effect of the curriculum on physician empathy or compassion.[32] We included outcomes measured from any perspective, including physician self-assessment as well as assessment by patients, standardized patients, or third party observers. We did not exclude studies based on language or publication type or date. We excluded secondary reports of previously published trials, reviews, correspondence, and editorials; however, we screened the reference lists of review articles to identify further studies for inclusion.

### Study selection and data abstraction

As described in our previously published protocol two independent reviewers screened the titles and abstracts of identified studies for potential eligibility. After completion of the relevance screen, the two reviewers compared exclusion logs to determine whether there was disagreement and used the Kappa statistic to quantify the inter-observer agreement. In cases of disagreement, the full text was reviewed for inclusion. For all studies deemed potentially relevant the full manuscripts were reviewed for inclusion. Two reviewers independently abstracted data on all study populations, interventions tested, outcome measures, and effect of interventions on outcome measures compared to control groups, using a standardized data collection form. Any disagreements in these processes were resolved by consensus with a third reviewer.[32]

### Assessment of study bias

Study quality was assessed using the Cochrane Collaboration’s tool for assessing the risk of bias evaluating six domains (selection, performance, detection, attrition, reporting, and other biases).[33]

### Analysis

We performed a primarily qualitative analysis of the literature in accordance with the recommended methodology for qualitative reviews published in the Cochrane Handbook.[33] In table format, stratified by individual publication, we collated and summarized the following: (1) study design; (2) population sampled (i.e. medical student, resident, attending physician); (3) sample size; (4) specific skills (e.g. identifying compassion opportunities) and behaviors [both verbal (e.g. compassionate statements) and non-verbal (e.g. eye contact, body position)] taught by the curriculum; (5) training methods utilized (e.g. lecture, small groups sessions, simulated experiential learning); (6) assessment methods for outcome measures; and (7) effect of curriculum on outcome measures compared to control groups. We determined the interventional curriculum of each study to be effective if the study identified a statistically significant difference in an empathy or compassion outcome measure in favor of the study curriculum group compared to the control group.

We were unable to use a meta-analytic approach to quantitatively analyze the data secondary to the heterogeneity in both interventions and outcome measures.

### Deviations from previously published protocol

We did not make any amendments to our original protocol.

## Results

### Search and selection

**Fig 1:**
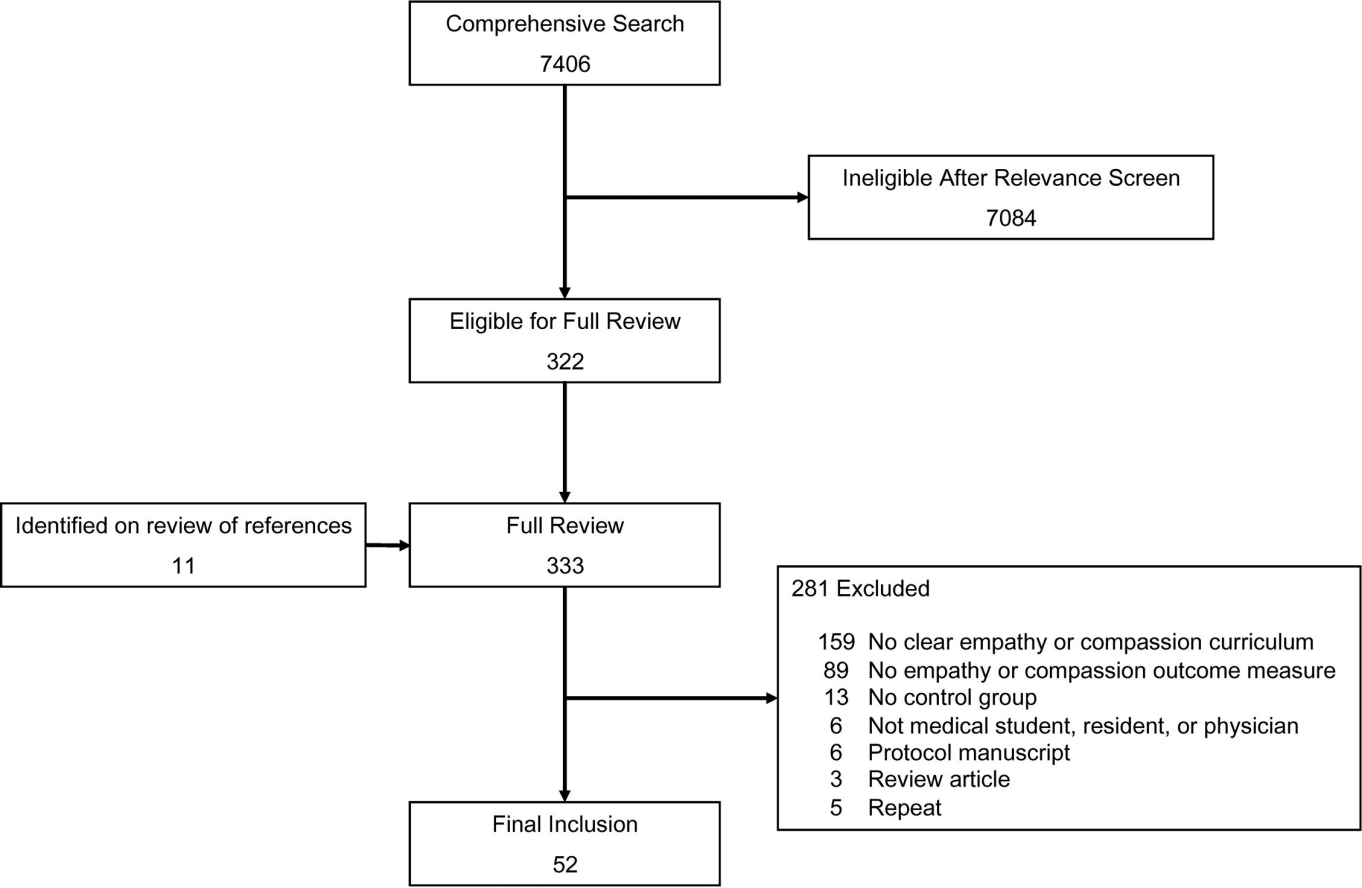
Search, inclusion, exclusion, flow diagram.

The initial database searches identified 7,406 potential articles. The majority of these studies were excluded (7,084) during the relevance screening (Fig 1). Studies were excluded during the relevance screening secondary to (1) no empathy or compassion training curriculum, (2) missing control arm, or (3) study population did not include medical students, residents, or physicians. Interobserver agreement for the relevance screening was excellent (κ = 0.90). On review of references we identified 11 additional potential articles. A full manuscript review was performed on 333 papers, resulting in 52 papers included for final analysis with a total of 5,316 subjects.

### Study characteristics

**Fig 2:**
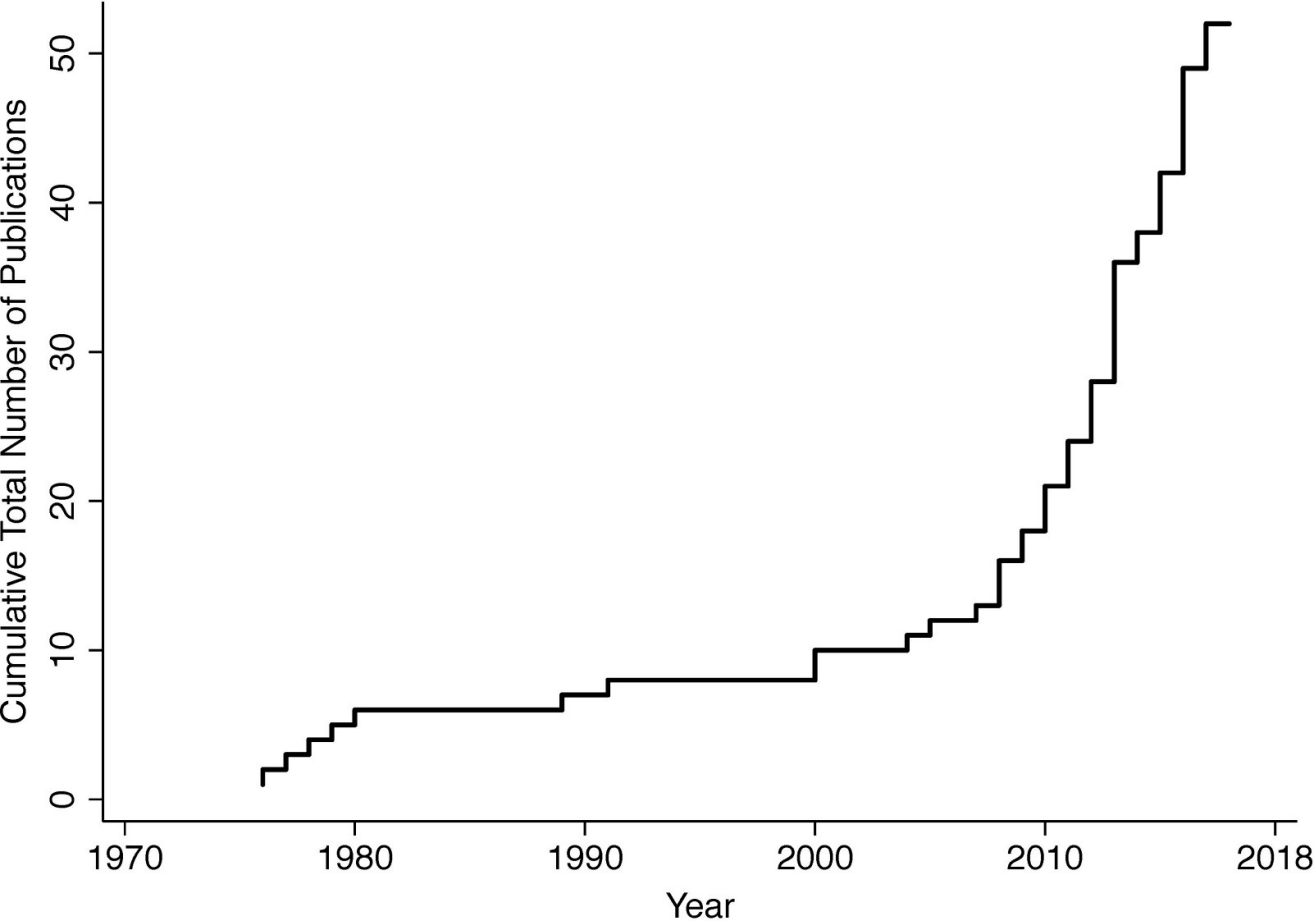
Cumulative number of included publications over time (in years).

The 52 studies were published over 42 years (1976–2018). Study characteristics for all 52 studies are displayed in Table 1. The majority of studies (54%) were published in the last five years (Fig 2). The most common study design was before/after [44% (23/52), n = 1,977], followed by randomized control trial [29% (15/52), n = 1,286], and prospective cohort study [27% (14/52), n = 2053]. The Cochrane tool for assessing risk of bias identified some concern for risk of bias for all included studies (i.e. “high risk” or “unclear risk”) (S1 Table). There were no adverse events related to the study interventions reported.

**Table 1:** Characteristics and outcomes of included studies.

Reference	Year	Study design Population n (intervention): n (control)	Skills/behaviors taught	Training methods	Effect of intervention on outcome measures compared to control
Bentley, et al[37]	2018	Before/after Psychiatry residents Self control (7)	Mindfulness skills (observing, describing, non-judging, non-reacting, and acting with awareness) Reflective listening (express a non-judgmental understanding of another’s experience)	Eight weekly 1.5 hour sessions Classroom didactics Handouts Video demonstration Practice exercises and brief role plays	At completion of the program: Increase in HRQ scores (pre-intervention mean = 1.21; post-intervention mean = 1.97; p = .02) Non-statistically significant decrease in MBI-HSS subscales (emotional mean change from 27.83 to 25.83, p = .509; depersonalization mean change from13.5 to 12.83, p = .632; personal accomplishment mean change from 38.33 to 36.83, p = .382)
Dotters-Katz, et al[38]	2018	Prospective cohort study with matched controls Obstetrics and Gynecology and Internal Medicine residents 10:10	Handling difficult communication Attentive observation (focusing awareness on another person)	Two 2 hour sessions Classroom didactics Roleplaying	60 Days after training: Decreased burnout scores (−3.1 vs. 2.5, P = 0.048) Trend toward an improved self reported compassion score (4.4 vs.−0.6, P = 0.096) No change in the PMI
Wündrich, et al[39]	2017	RCT Third year medical students total n = 158	Active listening Understanding the situation Understanding the problems Understanding feelings Explanation (of the illness, drugs, and so on) Shared decision-making Communicating hope Being competent Verbal expression Non-verbal expression Degree of coherence in the interview.	Two 2.25 hour sessions Simulated psychiatric patients Followed by structured feedback from standardized patient	Increased total empathy score rated by third party experts during OCSE (3.9±0.5 vs. 3.4± 0.5, p < 0.001) Increased total empathy score rated by simulated patients during OCSE (4.0±0.5 vs. 3.6±0.5, p < 0.001) No difference in JSPE
Schweller, et al[40]	2017	Before/after 1^st^ year medical students Self control (166)	Professional identity formation in a positive way Sharing someone’s pain is not painful and sharing experiences of patients allows self-reflection, which invariably leads to personal growth and development	Four month course Interviews with physicians and patients who shared real experiences Videos and live acting of simulated bad patient interactions followed by discussions	Increase in JSPE at the end of the course (pre 117.9 vs. post 121.3, p < .001)
LoSasso, et al[41]	2017	RCT 3^rd^ year medical students 38:32	SALTED mnemonic (Set-up, Ask, Listen, Type, Exceptions, and Documentation)	One hour session Classroom didactic and four brief roleplaying scenarios	No difference in the JSPE Increase in the JSPPPE as rated by third party observers (4.4 vs. 3.9, p = 0.02), but not standardized patients (3.5 vs. 3.1, p = 0.07)
Ruiz‐Moral, et al[42]	2017	Before/after 3^rd^ year medical students Self control (115)	Ability to detect and explore relevant patients’ “contextual and emotional clues” in a medical consultation Ability to tailor their empathic response depending on the clue	Six week course Demonstrative and small group work sessions Discussion of personal illness experience Workshops with simulated patients Peer group practice and reports Interviews with standardized patients	As rated by third party observer, statistically significant improvement in: 1. Non-verbal language (eye contact, verbal reactivity, …) 2. Discovering Ideas (points of view) fears and experiences 3. Empathy and support 4. Balanced open-ended and closed questions 5. Discourse facilitation 6. Grasping and following clues As rated by standardized patients statistically significant improvement in: 1. I felt the student was interested in me as a person, I felt supported 2. I felt it easy to speak and explain 3. I expressed my points of view
Buffel du Vaure, et al[43]	2017	RCT 4^th^ year medical students 155:144	Balint group training: method of exploring the dynamics of patient interactions, and gaining insight into personal reactions to patients, in an effort to more effectively meet the biopsychosocial needs and challenges of patients.	7 sessions of 1.5 hour small group sessions, over 3 months	Increase in JSPE one week after last session (112 vs.108, p = 0.002) No difference in the CARE measure as rated by standardized patients
Zazulak, et al[44]	2017	Prospective cohort study Family medicine and obstetrics and gynecology residents 15:20	Insight into assumptions, judgments and biases often made Understanding of patients’ non-verbal cues Enhance tolerance of ambiguity and consider how personal experiences bias observations and interpretations	Four 3 hour *Art of Seeing* sessions Introduction to formal art analysis Introduction to symbols in art Mindful (dance) movement workshop Looking beyond: introduction to conceptual and contemporary art	No difference in the IRI or compassion scale
Delacruz, et al[45]	2017	Before/after 1^st^ year pediatric and internal medicine-pediatrics residents Self control (33)	Introduce Initiation of conversation Listening and non-verbal skills (i.e. not distracted) Acknowledge emotional state of patient/parent Further explore and validate emotion or parent/patient experience Apologize (if needed) Verbal commitment to next steps Leave positive emotional wake (i.e. confirm agreement with plan and allow questions)	1.5 hour session Didactic session Small group discussion Interactive panel session with parents of patients	Increased empathy as third party observers (HEAT score 23.15 vs. 25.36, p < 0.001)
Flint, et al[46]	2017	Before/after Pediatric residents Self control (42)	Managing emotional responses: naming the emotion and exploring the cause of the emotion Expression of empathy: appropriate verbiage to use and to avoid	One 3-hour small group workshop	Increased self-assessed confidence in expressing empathy.
Ditton‐Phare, et al[47]	2016	Before/after Psychiatry residents Self control (30)	Encourage expression of feelings Acknowledge Normalize Validate Ask open questions Maintain eye contact Allow time to integrate Offer tissues Provide hope and reassurance	45 min classroom didactic session Followed by 2 hour small group role-play session	Non-statistically significant improvement in empathetic communication as rated by third party observer (4.18 vs. 3.30, p = 0.086)
Boissy, et al[48]	2016	Prospective cohort study Attending physicians 443:478	Support “I’m here for you. Let’s work together.” Acknowledge “This has been hard for you.” or “I’m sorry for the wait. I value your time.” Patient’s perspective (Occupational, interpersonal, intrapersonal) “How does it disrupt your daily activity?” or “How does it impact your functioning?” Ideas “What do you think is wrong?” Assess …what the patient knows about diagnosis & treatment …how much & what type of education is desired …patient treatment preferences …health literacy …patient understanding & emotional reaction to information provided Validate “Most people would feel the way you do.” or “Anyone in your position would feel upset.” Emotion naming “You seem sad.” Expectations “What are you hoping I can do for you today?” Worries “What concerns you most about it?” Inform …about diagnosis, treatment options, professional opinion	One day course Classroom didactic session, live or video-based skill demonstrations, and small group skills practice sessions	Higher overall mean CGCAHPS scores (92.09 vs. 91.09, p = 0.03) No difference in overall mean HCAHPS scores (83.95 vs. 82.73, p = 0.24) Increased JSPE pre- vs. post-intervention (124.1 vs. 116.4, p < 0.001)
Foster, et al[49]	2016	RCT Medical students 35: empathy feedback after virtual patient interaction 18: virtual patient with back story and no feedback 17: virtual patient with no back story and no feedback (controls)	Identifying compassionate opportunities and improving type of response Response type in descending order from best to worst: Shared feeling or experience Conformation Acknowledgment with pursuit Acknowledgment Implicit recognition Perfunctory recognition Denial of patient perspective	Empathy-feedback page for student to review at the end of the virtual patient interaction with coded empathic responses and potential response alternatives	Higher empathy among empathy feedback group during standardized patient interview as rated by third party observer compared to backstory and control groups; however only statistically significant compared to backstory group. Standardized patients rated increased empathic statements, appearing warm and caring, and forming rapport among empathy feed group compared to backstory and control groups.
Orloski, et al[50]	2016	Prospective cohort Emergency medicine physicians 574:793 (patients evaluated by standing vs. seated physicians)	Physicians educated to sit during patient interview	Folding stool provided to the emergency department Educational campaign to sit during patient interview	Patients were more likely to select “strongly agree” (highest mark) for physician politeness, caring, listened, informed, and time spent
Duke, et al[51]	2015	Before/after 3^rd^ year medical students Self control (259)	Self-awareness to help manage the “hidden curriculum”	Small groups meet every 8–12 weeks on a social networking platform For each session students wrote a brief self-reflection about a meaningful experience, which were used for discussions.	Increase in the GRAS (94 vs. 91, p < 0.001) No statistically significant difference in the JSPE (115 vs. 113, p = 0.07)
Lusilla‐Palacios, et al[52]	2015	Before/after Rehabilitation staff working in spinal cord injury unit Self control (45)	Motivational interviewing (listen and reflect back so that the patient can hear their thoughts and motivations expressed back to them)[53]	Baseline assessment (months 1–12) Focus groups (months 13–14) Two day classroom didactic session (12 hour total during months 15–17) Coaching was delivered on demand, individually, or in small groups, in 60 min sessions (months 18–23) Followed by 2 hour voluntary review session (months 25–30)	No difference in the JSPE
Potash, et al[54]	2014	RCT 3^rd^ year medical students 48:58 (control: clinical case problem solving session)	Creative engagement (developing multiple perspectives that can help to provide insight and awareness as to how a patient experiences pain and suffering)	3 hour workshop Wrote a poem about a memory witnessing a patient in pain or suffering. Created a drawing or painting based on the poem Small group session discussing art Wrote a reflective essay about how the art making experience affected their understanding of patients	No difference in JSPE
Nasr, et al[55]	2014	RCT 1^st^ year psychiatry residents 7:7 (group 1 attended both days and group 2 watched a video of day one and attended day two).	Clinical setting and basic communication skills in therapeutic relationship Obtaining and delivering information to the patient	Two six hour days Day 1: Classroom didactic session and rfoleplaying Day 2: Roleplaying and group discussion	Group 1 had an increase in OAE between pre- and 3 months post-intervention as rated by third party observer. No differences in JSPE or JSPPPE
Williams, et al[56]	2014	Before/after 1^st^ year medical students Self control (122)	Reflecting on patient interactions What do you think the needs of the patient were? Do you think the patient’s needs were met in this clinical interaction? What empathetic behaviors did you see or observe in this scenario? What was the impact of this behavior on interaction between the patient and doctor?	2 hour interactive empathy workshop was based on a 20-min DVD simulation	Increased JSPE between pre- and 5 weeks post-intervention (118 vs. 112 p < 0.001)
Airagnes, et al[57]	2014	Prospective cohort study 4^th^ year medical students 34:129	Balint group training: method of exploring the dynamics of patient interactions, and gaining insight into personal reactions to patients, in an effort to more effectively meet the biopsychosocial needs and challenges of patients.	10 two-hour weekly sessions Small groups sessions discussing experiences with patients	No difference in IRI between groups
Schweller, et al[58]	2014	Before/after 4^th^ year medical students Self control (124) 6^th^ year medical students Self control (123)	Identifying patient feelings of the patient about the disease, such as fear, guilt, anger, and abandonment, and the feelings of the doctor towards the patient	Four weekly sessions over 30 days Standardized patients followed by a small group debriefing	4^th^ year students: increase in JSPE (121 vs. 116, p < 0.001) and increase in IRI (67 vs. 65, p = 0.003) 6^th^ year students: increase in JSPE (124 vs. 117, p < 0.001) and increase in IRI (66 vs. 66, p < 0.001)
Graham, et al[59]	2014	Before/after Pediatric and family medicine residents Self control (79)	Individualized patient management Eliciting the patient perspective to allow structured individualized care	40 min classroom didactic lead by trained patients 20 min question and answer session	Increase in 10-item subscale of JSPE (66 vs. 63, p = 0.025)
Bays, et al[60]	2014	Before/after Internal medicine residents, internal medicine subspecialty fellows, and nurse practitioner students Self control (145)	NURSE: Naming emotion expressing Understanding of a patient’s feelings or situation showing Respect or praise for the patient articulating Support for the patient Exploring the patient’s emotional state	Eight four-hour sessions over a month Brief didactic overview Skills practice using a standardized patient Reflective discussions	Increase in all NURSE subscales except exploring the patient’s emotional state, as rated by third party observer
Tang, et al[61]	2014	Before/after Oncologists Self control (28)	Balint group training: method of exploring the dynamics of patient interactions, and gaining insight into personal reactions to patients, in an effort to more effectively meet the biopsychosocial needs and challenges of patients.	Three 0.5-hour lectures Two fishbowls Four case discussions in small group One discussion on feedback	No difference in the JSPE
Yang, et al[62]	2013	Before/after Clerks and interns Self control (110)	Understand in the context of patients’ beliefs, and family and cultural values Understand the personal health care should include physical, mental, emotional and social concerns	4 hour small group session discussing art	No difference in the JSPE
Gibon, et al[63]	2013	RCT with waitlist Radiotherapy team members 65:31	Assessment of patients’ concerns and needs and to improve the information and support given to the patients Facilitate communicate with team members and patients	16-h patient-oriented communication skills training module followed by a 22-h team-resource-oriented communication skills training module over four months Small groups	Increased rate of empathy statements as assessed by third party observer (Empathy statement: showing an understanding of the patient’s emotional or physical state) RR: 4.05 (95% CI 1.09 to 15.11)
Johnson, et al[64]	2013	RCT with waitlist Senior cancer health professionals 12:9	Distinguishing positive and negative communication behaviors Strategies for handling difficult communication situations Understanding emotional impact of communication	3 day course Roleplaying and feedback	No difference in patient assessment of compassion as measured by the CARE measure one month after training
Blanco, et al[65]	2013	Before/after Internal Medicine, Psychiatry, Family Medicine, Internal Medicine-Pediatrics, Obstetrics and Gynaecology, Pediatrics, and Surgery residents Self control (41)	Direct and focus one’s attention Recognize non-verbal cues Actively listen Show interest in the whole person Nonjudgmentally value each person Ask about emotions, concerns, and distress Respond to emotions, concerns, and distress Share information and decision making Demonstrate trustworthiness	Half-day core workshop with reflective exercises, case discussions and role-play. Journal writing Four 1-hour follow up meetings	No statistically significant change in interpersonal and communication skills performance on a standardized patient encounter as graded by third party observer. No statistically significant change in JSPE.
Riess, et al[66]	2012	RCT Medicine, general surgery, anesthesia, psychiatry, orthopedic, and ophthalmology residents 54:45	Provide the scientific foundation for the neurobiology and physiology of empathy training Understand the physiology of emotions during typical and difficult patient–physician interactions Decoding subtle facial expressions of emotion Empathic verbal and behavioral responses	Three 60-minute modules over 4 weeks	Increased patient assessment of compassion as measured by the CARE measure one month after training (CARE measure change 0.7 vs. -1.5, p = 0.04) No difference in JSPE (JSPE change 1.2 vs. -1.1)
Cinar, et al[67]	2012	Before/after Emergency medicine residents Self control (20)	Understand empathy, communication, and relationships between the patient and health care team. Understanding dominant, passive, and aggressive communication types Verbal and nonverbal communication Active listening, recognizing and understanding feelings, expression of feelings and thoughts, identification with others, and establishing empathy	Six weekly sessions involving classroom didactics, case examples with discussion, and role playing	No change in the Empathy Quotient (29.5 to 30.7, p = 0.1) Increase in respect, kindness, and understanding as assessed by patients [90.3 ± 10.8 vs. 94.1 ± 16.5 (p< 0.01)]
Ozcan, et al[68]	2012	Before/after Medical and nursing students Self control (257)	Understanding empathetic tendency: Exploring Clarifying Sequencing Encouraging description of perceptions Reflecting Focusing Feedback Verbalizing the implied Attempting to express feelings Restatement Paraphrasing Accepting/offering general leads Giving broad openings	Five 2-hour weekly didactic sessions	Increase in the Empathic Communication Skill Scale and the Empathic Tendency Scale
Lim, et al[69]	2011	Prospective cohort study 5^th^ year medical students (intervention, 2010 students; control, 2009 students) 77:72	Connect with patients: listen to what they are saying, observe their body language, pick up interpersonal cues, and improve interpersonal and interactive skills	One hour didactic session and role playing	Increased JSPE (p < 0.001)
Tulsky, et al[70]	2011	RCT Oncologists 24:24	Principles of effect communication Recognizing empathic opportunities Responding to empathetic opportunities Conveying prognosis Responding to difficult questions	CD-ROM training program on communication skills that was tailored with exemplars from their own audio-recorded clinic visits	Increase in number of empathetic statements in response to empathetic opportunities during patient encounters (0.7 vs. 0.4, p = 0.024) Increase in patient perceived trust scale No change in patient perceived empathy No change in patient, “perceived belief that the oncologist cared about the patient,” or “perceived belief that the oncologist understood the patient as a whole person
Riess, et al[71]	2011	Before/after Otolaryngology residents Self control (11)	Physiological awareness and regulation of patient-physician interactions	Three 90-min sessions over 6 weeks. Videos display real-time physiological responses for both members of the patient-physician interactions, allowing observers to see the degree to which patient and physician are physiologically concordant or discordant with one another	No change in self reported BEES (50.2 vs. 45.2, p = 0.26) or JSPE (114.3 vs. 110.1, p = 0.19) No change in patient assessment of compassion as measured by the CARE measure (40.0 vs. 37.7, p = 0.31)
Cahan, et al[72]	2010	Prospective cohort study Medical students Pilot one 48:49 Pilot two 44:44	Define communication strategies that families interpret as a “caring attitude” Speak with and calm angry patients Deliver bad news in a caring manner	Two hour session Classroom didactic followed by standardized patients and feedback	Pilot one: no change in 5-point empathy score (2.85 vs. 2.84, p = 0.94) Pilot two: increase in 5-point empathy score (3.45 vs. 2.32, p < 0.001)
Sripada, et al[73]	2010	RCT Psychiatry residents 6:6	Empathetic accuracy	Mean 13.77 therapy sessions Resident and patient compared Global Assessment of Functioning scale scores	Improved empathetic accuracy and higher Barrett-Lennard empathy subscale score as assessed by patient.
Ghetti, et al[74]	2009	Before/after Obstetrics and gynecology residents Self control (17)	Balint group training: method of exploring the dynamics of patient interactions, and gaining insight into personal reactions to patients, in an effort to more effectively meet the biopsychosocial needs and challenges of patients.	Two 1-hour small group sessions	No change in JSPE 12-months after intervention
Bonvicini, et al[75]	2009	RCT Primary care physicians 79:76	‘‘4Es” (Engage, Empathize, Educate and Enlist) Motivational interviewing Understand the nature of interpersonal difficulties between clinicians and patients Recognize and assess tensions in relationships Acknowledge problems, Discover meaning, and showing compassion	3 six-hour monthly sessions Didactic and experiential teaching modalities Individual coaching Skills practice sessions	Video-taped patient encounters graded by third party observer: increase in the ECCS and GRS
Shapiro, et al[76]	2009	RCT with waitlist 1^st^ year medical students 38:41	Engage patient in a conversation Maintain a conversation Understand patient perspective Accurately track the emotional state of the patient Express care and concern without intrusiveness or use of platitudes Do all of the above without negating, belittling, or being controlling Elicit relevant information in an efficient manner (e.g., stay on topic) Explain and describe clearly and succinctly	Weekly meetings with patients on a one-to-one basis for four months while receiving group supervision and feedback from a faculty psychiatrist	No difference in Self Assessment of Interpersonal Competence Questionnaire or the Standardized patient assessment using the Interpersonal Skills Rating Scale. Improved score on the written responses to the Staff-Patient Interaction Rating Scale, as graded by third party reviewer. (2.29 vs. -0.68, p = 0.038)
Fernandez‐Olano, el al[77]	2008	Prospective cohort 2^nd^ year medical students and family medicine residents 128:75	Communication skills: cordiality, respect, assertiveness, controlled reactions, precision, active listening, two-way communication and empathy Understand the thoughts, emotions, and behavior of another Formulating empathetic phrases and non-verbal expressions	25-hour workshop over 5 days Small group sessions with exercises, analysis of video recordings, and role-playing	Increase in JSPE among intervention (125.1 vs. 119.5, p < 0.001), but not controls (119.1 vs. 118.2, ns).
Dow, et al[78]	2007	Prospective cohort study Internal medicine residents 14:6	Insight into patient behavior Active listening Listening for subtext, listening for values and strengths, making links to one’s own experiences, and strategies for acknowledging the patient’s feelings Skills in physical expressiveness, body language, and vocal presence	Four 90-minute classroom and small group workshop sessions in the Department of Theater	Increased empathetic communication, relating to the listening, nonverbal communication, respect for dignity, and overall impression. No change in verbal communication Assessed by third party observer
Cataldo, et al[79]	2005	Prospective cohort study Family medicine residents 74:40	Balint group training: method of exploring the dynamics of patient interactions, and gaining insight into personal reactions to patients, in an effort to more effectively meet the biopsychosocial needs and challenges of patients.	Once a week for an hour over 2 years	No difference in JSPE (119.4 vs. 116.7, p = 0.25)
Shapiro, et al[80]	2004	RCT with waitlist 1^st^ year medical students 10:9	Understanding different points of view, including those of physicians, patients, and family members	Eight small-group reading and discussion sessions, for 1 hour twice monthly	No difference in the ECRS (pre = 92.3 vs. post = 94.6; p = 0.27) Increase in the BEES (pre = 57.0, post = 68.9; p < 0.01)
Roter, et al[81]	2004	Before/after Pediatric residents Self control (28)	Listening more ⁄talking less Data gathering techniques using open-ended questions to probe patient’s knowledge, perceptions of care, treatment preferences, and lifestyle and psychosocial issues Responding to the parent⁄ guardian’s emotions; Building an active partnership for problem solving related to the therapeutic regimen.	One-hour didactic and role-playing practice session One-hour reviewing coded videotape (recording of previous interaction with standardized patient) within an interactive CD-ROM platform focusing on areas of communication related	Increase in the expression of empathy as rated by a third party observer using Roter Interaction Analysis System
Winefield, et al[82]	2000	Before/after 1^st^ year medical students Self control (107)	Introduce Non-verbal attentiveness Active listening Information-gathering using open-ended questions, Following leads from the patient Making empathetic responses Using appropriate language complexity	Didactic lecture Videotape and written handouts Two 1.5-h workshops, a week apart, practicing interviewing techniques with feedback and video recording of interview	Improvement in investigator developed written empathy test (score range 0–40) [pre: 9.97 vs. post: 14.44]
Moorhead, et al[83]	1991	Before/after 4^th^ year medical students Self control (63)	Holism Patient-centeredness Empathetic active listening	3 hour small group session 1.5 hour communication training from a social worker 10 hours practicing interviewing real and standardized patients	No change in the Empathy Rating Scale as measured by third party raters (12.6 vs. 12.8)
Kramer, et al[84]	1989	RCT 5^th^ year medical students A. No workshop (10) B. Tutors participated in workshop (10) C. Students participated in workshop (10) D. Students and tutors participated in workshop (10)	Verbal explanation Small talk Listening Calming Empathetic response Encouragement Questioning Nodding Smiling Laughing Eye contact Supportive touching	Ten 90-minute session held twice weekly. Role playing and facilitated discussions	Third party observers counted number of empathetic supportive behaviors during medical interviews by students. Increase in supportive behaviors among groups C and D compared to A at 6 and 12 months after workshop.
Poole, et al[85]	1980	Prospective cohort 2^nd^ year medical students 25:20	Commercial training program: "Tune-In, Empathy Training Workshop”[86]	Eight 1.5 to 2 hour audiotape-led sessions	Improvement in the Accurate Empathy Scale as rated by a third party observer during a patient interview three years after the intervention compared to pre-intervention, as well as compared to controls.
Junek, et al[87]	1979	Before/after 1^st^ year psychiatry residents Self control (5)	Carefully listen Note incongruities between patient’s affect, words and body posture Avoid assumptions State the nature of relationship with the patient Do not fear silences Make statements in lieu of questions Be aware of own emotions Stay in the here and now with the patient	Twelve 1.5-hour weekly sessions Didactic class room sessions Practice interviews with real patients followed by feedback	Improvement in all four components of the Modified Barrett-Lennard Relationship Inventory (empathy, congruence, level of regard, unconditionally) as rated by third party observer.
Sanson-Fisher, et al[88]	1978	Prospective cohort 2^nd^ year medical students 112:23	Commercial training program: "Tune-In, Empathy Training Workshop”[86]	Eight 1.5 to 2 hour audiotape-led sessions	Improvement in the Accurate Empathy Scale as rated by a third party observer during a patient interview compared to pre-intervention, as well as compared to controls.
Fine, et al[89]	1977	Prospective cohort 1^st^ year medical students 20:23	Avoidance of responses known to block further communication Uses responses known to increase trust and openness	Eight 1.5 weekly sessions Roleplaying	Improvement in Traux Accurate Empathy Scale on written responses to patient problems as rated by third party reviewer
Pacoe, et al[90]	1976	Prospective cohort 1^st^ year medical students 13:7	Develop responses in which the levels of the “core” therapeutic qualities could be increased: example, “I am with you.”	Sixteen 2.5 hour weekly sessions Roleplaying discuss real personal issues followed by group feedback	Improvement in the Wells Empathetic Communication test (handwritten responses to video excerpts grader by third party reviewers) and the Index of Facilitative Discrimination (multiple choice test to identify the most empathetic responses).

BEES, Balanced Emotional Empathy Scale; CI, confidence interval; CGCAHPS, Clinician and Group Consumer Assessment of Health care Providers and Systems; ECRS, Empathy Construct Rating Scale; GRAS, Groningen Reflection Ability Scale; HCAHPS, Hospital Consumer Assessment of Health care Providers and Systems; HEAT, hear, empathize, apologize, take action; HRQ, The Helpful Responses Questionnaire; IRI, Interpersonal Reactivity Index Empathy Scale; JSPE, Jefferson Scale of Physician Empathy; JSPPPE, Jefferson Scale of Patient Perceptions of Physician Empathy; MBI-HSS, Maslach Burnout Inventory—Human Services Survey; OAE, objective assessment of empathy; OCSE, objective clinical structured examination; PMI, Psychological Medicine Inventory; RR, relative risk.

### Study populations

Forty-six percent (24/52, n = 3120) of studies tested the training curriculum among medical students and 38% (20/52, n = 882) tested the curriculum among residents. Only eight studies (15%, n = 1314) tested the curriculum among practicing physicians.

### Training methods

Duration of the included training curricula varied considerably from a single one-hour session to multiple sessions over three years. The majority of the study curricula involved more than one session [75% (39/52), n = 3323). The majority of the study curricula incorporated small group sessions as a part of the curriculum [63%, 33/52, n = 3791] and 46% (24/52, n = 2544) incorporated didactic lectures. Thirty (58%, n = 2679) studies incorporated practicing learned skills through role-playing (16 studies, n = 1246), standardized patient interviews (9 studies, n = 1118), or real patient interactions (6 studies, n = 343). One included study incorporated both role-playing and standardized patient interviews (n = 28). Four studies (n = 386) incorporated video recording of interviews, on which subjects reviewed and received feedback.

### Outcome measures

The majority of studies tested the effects of the training curriculum on self-assessed outcomes [56% (29/52), n = 3643] (i.e. trainees assessment of their own empathy or compassion). The most commonly used self-assessment outcome measure was the Jefferson Scale of Empathy [72% (21/29), n = 3258). Twenty-five studies (48%, n = 2002) measured empathy or compassion as rated by a third party observer, seven studies (13%, n = 805) as rated by standardized patients, and eight studies (15%, n = 1132) as rated by actual patients. The majority of studies used a previously validated measurement tool [73% (38/52), n = 4342], while 17 studies (33%, n = 1098) incorporated a new measurement tool. Only two studies evaluated long-term effects of the training curriculum (i.e. at 12 months after completing training).[74, 84]

### Study results

**Fig 3:**
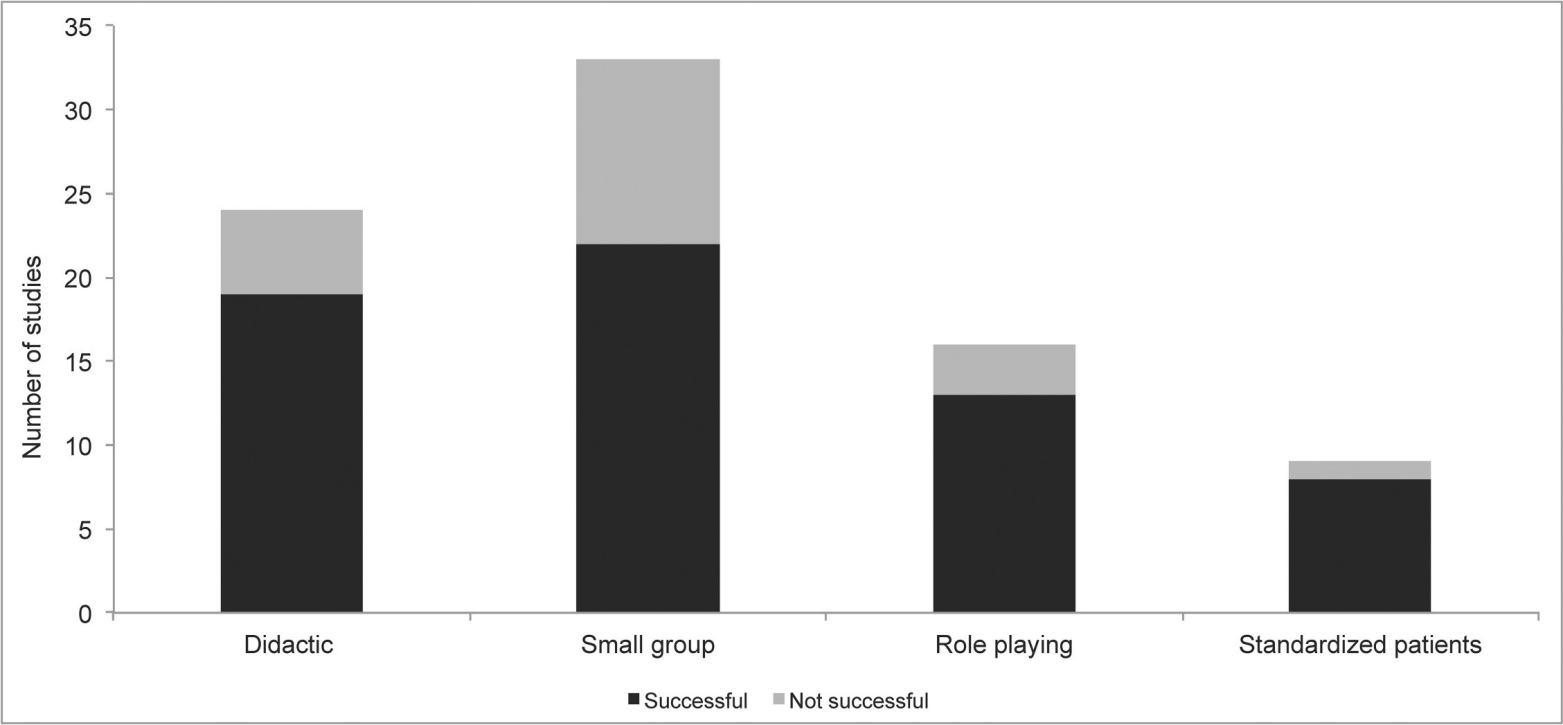
Frequency of successful and non-successful studies by training method.

The majority of studies found the tested training curriculum improved physician empathy or compassion as measured on at least one outcome measure [75% (39/52), n = 4532]. Success rates among studies involving medical students, residents, and physicians were, 87% (21/24), 65% (13/20), and 63% (5/8) respectively. Success rates among studies using self-assessment outcomes, third party raters, standardized patient raters, and actual patient raters were, 45% (13/29), 88% (22/25), 57% (4/7), and 75% (6/8) respectively. We found training methods involving actual patients (six studies), as well as video recording of interviews (four studies), had the highest success rate with 100% of these curricula demonstrating improvement on at least one outcome measure. Success rates for other training methods are displayed in Fig 3. We found 77% (30/39) of curricula involving more than one session had improvement on at least one outcome measure compared to 69% (9/13) of curricula involving a single session.

### Clinical skills and behaviors

All study curricula incorporated teaching some aspect of taking time to listen and/or having awareness of the patient’s emotional state. Skills and behaviors that demonstrated an increase in real patient perception of compassion included (1) sitting (versus standing) during the interview;[50] (2) detecting patients’ facial expressions and non-verbal cues of emotion;[66] (3) recognizing and responding to opportunities for compassion;[70] (4) non-verbal communication of caring [i.e. employing non-verbal caring behavior (e.g. body position facing the patient, eye contact, tone of voice, and appropriate hand and arm movements), as opposed to avoidant or aggressive behavior];[67] (5) incorporating statements of support (e.g. “I’m here for you. Let’s work together”), worry (e.g. “What concerns you most?”), acknowledgement (e.g. “This has been hard for you”), patient’s perspective (e.g. “How does it disrupt your daily activity?”), emotion naming (e.g. “You seem sad”), and validation (e.g. “Most people would feel the way you do”).[48]

Of the two studies that measured outcomes out to 12 months, one curriculum incorporated Balint group training (i.e. a method of exploring the dynamics of patient interactions, and gaining insight into personal reactions to patients, in an effort to more effectively meet the biopsychosocial needs and challenges of patients), and did not find a difference in Jefferson Scale of Physician Empathy at 12 months.[74] The second curriculum taught specific skills including verbal explanation, small talk, listening, calming, compassionate response, encouragement, questioning, nodding, smiling, laughing, eye contact, and supportive touching, and found an increase in supportive behaviors at 12 months as measured by a third party observer.[84] Table 1 displays the clinical skills and behaviors taught among the included studies, along with outcome measures and results.

## Discussion

In this report, we collated the current biomedical literature on empathy and compassion training curricula for physicians and physicians-in-training. Our objective was to qualitatively describe the specific skills and behaviors that have previously been demonstrated to improve physician empathy and compassion, and the methods of training that are most effective at transferring this knowledge to the learner.

Consistent with previous reports, we found that among the 52 studies meeting criteria for inclusion the preponderance of evidence indicates that training curricula are effective for enhancing physician empathy and compassion. This report further advances this field of research in that we have tabulated the specific skills and behaviors, which have been demonstrated to enhance (or failed to enhance) physician empathy and compassion. Thus, we have developed an evidence-based framework from which researchers and educators can develop and test future training curricula. Specifically, we identified the following behaviors may improve patient perception of provider empathy and compassion: (1) sitting (versus standing) during the interview; (2) detecting patients’ facial expressions and non-verbal cues of emotion; (3) recognizing and responding to opportunities for compassion; (4) non-verbal communication of caring (e.g. facing the patient, eye contact); and (5) verbal statements of acknowledgement, validation, and support. A possible common denominator among these interventions is assuring the patient of true physician presence and focus, and that they are not going through their current medical condition alone, but that they have the full attention and support of the physician.

This report found heterogeneity in the curricula studied, as well as outcome measures used to test the effectiveness of the curricula. Patient perspective of physician compassion has previously been demonstrated to be associated with improved clinical outcomes.[91–93] However, in this systematic review we only identified eight studies measuring physician empathy/compassion from the patient perspective. A commonality among these studies was a focus on taking time to be fully present, listening, and/or having awareness of the patient’s emotional state. Learning and incorporating such skills shifts the focus from narrow biomedical inquiry to knowing the patient as a whole person, which has been demonstrated leads to increased patient-reported trust in their provider.[70] Such trust has been demonstrated to improve compliance with prescribed therapies and has been suggested to improve clinical outcomes.[6] We also found that teaching providers specific skills and behaviors increased patient assessment of physician compassion. Thus, we propose the design of future curricula should include the training of nonverbal behaviors such as sitting, body position towards the patient, calm tone of voice, and eye contact. These results are consistent with previous evidence that physicians who sit during consultation are considered to be more compassionate compared to those that stand.[94] Similarly, Sherer *et al* found that observers rated psychology therapists who sit in close proximity to the patient (91 cm), in addition to provide consistent eye contact (90% of the time), as having more empathy, warmth, and genuineness compared to those that sat further (213 cm) and provided minimal eye contact (10% of the time).[95] Future curricula should also focus on educating providers on the importance of listening and identifying empathy and compassion opportunities, as well as provide guidance/examples on how to respond to these opportunities with statements of support, acknowledgement, and validation.[48] Finally, we propose that testing of these curricula should incorporate validated outcome measures, which measure actual patient perception of physician empathy and compassion, as opposed to standardized patients or third-party reported measures.[96] Given that the patient experience of compassion (or lack thereof) is likely what drives the association between physician compassion and clinical outcomes, future research should employ patient assessment of physician compassion for testing the effects of training curricula on clinical outcomes.

Importantly, burnout among physicians has a major economic toll on health care, as well as a major toll on the health of patients.[97] Burnout has been identified as a major public health issue, with recent reports identifying that approximately 50% of physicians are experiencing burnout.[98] There is now evidence that compassionate patient care may be beneficial for physicians. Specifically, compassionate patient care may enhance physician resilience and resistance to burnout.[99–101] Therefore, empathy and compassion training curricula may be an effective therapy to reduce physician burnout. Thus, in addition to measuring patient perspective of physician compassion, future curricula should also incorporate specific skills and behaviors demonstrated to improve physician self-assessment of empathy and compassion (Table 1).

This systematic review also identified methods of training that are most effective. The preponderance of evidence to date suggests that in addition to didactic lectures, incorporating a curriculum in which physicians can practice learned skills might be the best for enhancing physician empathy and compassion. Specifically, similar to medical training in which clinical skills are practiced in real time with actual patients and oversight by practicing physicians, incorporating similar methods to “practice” compassion in the clinical setting appears to be beneficial. Additionally, in certain clinical settings, and with patient consent, videotaping interactions with patients as a method to provide feedback on verbal and non-verbal behaviors appears to enhance compassionate behaviors. Of similar importance we identified curriculum and training methods that were not effective. We found studies that only incorporated didactic and/or small group sessions were the least likely to be effective (13/22). None of the four studies that focused on using art were found to improve self-reported empathy scores.[44, 54, 62, 65] In addition, only one of the five studies that focused on Balint group training had a positive effect on the reported outcome measure.[43, 57, 61, 74, 79]

We recognize there are important limitations of this systematic review to consider. First, all 52 included studies had some risk of bias according to the Cochrane Collaboration’s tool for assessing the risk of bias in clinical trials. Therefore, the results of the included studies must be interpreted with some caution. Second, there were varying educational scenarios, curricula studied, and outcomes measurements used, resulting in a high degree of heterogeneity. Therefore, we were not able to perform a quantitative meta-analysis to determine the effects of specific curricula on any particular clinical outcome. Third, there was a paucity of studies evaluating long-term outcomes. Thus, we are not able to determine if the effects of the tested curricula are sustained over time. However, one study found that teaching specific verbal and non-verbal behaviors resulted in increased supportive behaviors at 12 months after the training.[84] Fourth, there is significant overlap between the constructs of empathy and compassion,[5] and to date there is no agreed upon instrument to measure empathy and compassion in health care.[102] While the majority of the studies reported an outcome measure of empathy (48/52), it is possible that a component of compassion was also being measured. For example, the Consultation and Relational Empathy (CARE) measure is stated to measure empathy; however, one of the items of this measure specifically asks, “how was the doctor at showing care and compassion?” Thus, given the complex nature of the empathy/compassion relationship it is unlikely that the intervention curricula affected, or the outcome measures assessed, either empathy or compassion alone. Therefore, we believe it is not possible to precisely differentiate the two constructs in this report, and future research is required to further delineate and define the different effects of empathy versus compassion training. Fifth, none of the studies assessed direct clinical outcomes of patients, and as such it is not clear whether the observed changes in the indices of empathy or compassion training have meaningful implications for patient health outcomes. Sixth, although we searched the databases generally considered to be the most important sources to search,[33] and we did not exclude studies based on publication type, it remains possible that pertinent studies were conducted and either not published or not captured by our search strategy.

## Conclusion

In summary, current evidence suggests that training can enhance physician empathy and compassion. This report has collated the medical education literature on skills and behaviors that enhance physician empathy and compassion, and provides a framework from which researchers and educators can develop evidence-based curricula.

## Supporting information

S1 File PRISMA checklist. (DOC)

S1 Table Cochrane collaboration’s tool for assessing the risk of bias for each included article. (DOCX)
