# Supplementary material for: Curricula for empathy and compassion training in medical education: A systematic review
Source: PLoS One. 2019 Aug 22;14(8):e0221412. doi: 10.1371/journal.pone.0221412 (PMC6705835; doi:10.1371/journal.pone.0221412)
Supplement: S1 Table — (DOCX) [file pone.0221412.s002.docx]

**Supplemental Table 1**: Cochrane Collaboration’s tool for assessing the risk of bias for each included article

**Journal Article:**

Bentley PG, Kaplan SG, Mokonogho J. Relational Mindfulness for Psychiatry Residents: a Pilot Course in Empathy Development and Burnout Prevention. *Acad Psychiatry.* 2018.

| **Entry** | **Judgement** | **Support for judgement** |
| --- | --- | --- |
| Random sequence generation (selection bias) | High risk | Quote: “Limitations of this pilot include… lack of a control group”  Comment: No randomization - before/after survey trial. |
| Allocation concealment (selection bias) | High risk | Quote: “A total of seven PGY-1s (5 men; 2 women) at an academic medical center… before and after the course.”  Comment: No randomization - self control. |
| Blinding of participants and personnel (performance bias) | High risk | Quote: “A total of seven PGY-1s (5 men; 2 women) at an academic medical center...before and after the course.”  Comment: Unable to blind participation in the course. |
| Blinding of outcome assessment (detection bias) | High risk | Quote: “Limitations of this pilot include… reliance on self report measures.”  Comment: Unable to blind outcome assessment because they are self reports. |
| Incomplete outcome data addressed (attrition bias) | Low risk | Quote: “A total of seven PGY1s… completed the HRQ, MSI-HSS... before and after the course.”  Comment: No attrition. |
| Selective reporting (reporting bias) | Unclear risk | Comment: Study protocol not available. |
| Other Bias:  (social desirability bias) | High risk | Quote: “There may be some social desirability issues due to the intervention being taught by the researchers.”  Comment: May be limited because researchers are not attendings evaluating PGY-1s but there is still the potential for social desirability bias. |
| Other Bias:  (maturation bias) | High risk | Comment: Natural change/improvement in time may have lead to higher post test scores. No control group to compare. |
| Other Bias:  (Hawthorne effect) | High risk | Comment: The tendency to perform better when participating in an experiment may have lead to higher post test scores. |
| Other Bias:  (small sample size) | High risk | Quote: “A total of seven PGY-1s (5 men; 2 women) at an academic medical center.”  Comment: Small sample size increases the possibility that results were due to chance and limits generalizability. |

**Journal Article:**

Dotters-Katz SK, Chuang A, Weil A, Howell JO. Developing a pilot curriculum to foster humanism among graduate medical trainees. *J Educ Health Promot.* 2018;7:2.

| **Entry** | **Judgement** | **Support for judgement** |
| --- | --- | --- |
| Random sequence generation (selection bias) | High risk | Quote: “Residents were contacted by E-mail and asked to participate in the pilot curriculum. The first twelve residents to respond were invited to participate.” (and) “Control residents were selected by...program coordinators. The choice of controls was left to the discretion of the program coordinators with an attempt to match the number of participants and controls by the type of residency program and gender”  Comment: No randomization - matched controls. |
| Allocation concealment (selection bias) | High risk | Quote: “Baseline questionnaires were completed before the first session. Follow-up questionnaires were administered 60 days from the last curriculum session.”  Comment: No randomization - before/after with matched controls. |
| Blinding of participants and personnel (performance bias) | High risk | Quote: “Participants had to attend at least two of the three planned 2 h sessions to be included in the analysis.”  Comment: Unable to blind participation in sessions. |
| Blinding of outcome assessment (detection bias) | High risk | Quote: “The effectiveness of the curriculum was measured at baseline (before the first session) and at 60-day follow-up with questionnaire scores assessing… burnout, compassion … and ability to practice psychological medicine (Psychological Medicine Inventory [PMI]).”  Comment: Unable to blind outcome assessments as they are self reports. |
| Incomplete outcome data addressed (attrition bias) | Low risk | Quote: “Regarding the humanism sessions, twelve students expressed interested in the curriculum, but only ten completed it.”  Comment: While only 10 completed the session, all 10 filled out the questionnaires in addition to 10 controls. |
| Selective reporting (reporting bias) | Unclear risk | Comment: Study protocol not available. |
| Other Bias:  (social desirability bias) | High risk | Comment: Potential for learners to want to answer questions in a way that would be viewed favorably by others. |
| Other Bias:  (Hawthorne effect) | High risk | Comment: Students are aware of participation in a study which could affect their behavior and questionnaire responses. |
| Other Bias:  (small sample size) | High risk | Quote: “Only ten completed it.”  Comment: Small sample size increases the possibility that results were due to chance and limits generalizability. |
| Other Bias:  (chance bias) | High risk | Quote: “At a baseline...there was a trend toward higher burnout in the participant group.” (and) “...we compared each individual to herself and used mean differences to compare the two cohorts.”  Comment: With higher burnout in the participant group compared to control, there is more room for improvement in scores/greater mean differences. |
| Other Bias:  (other) | High risk | Quote: “A voluntary cohort is like to be more interested in medical humanism or perhaps more likely to be struggling with professionalism issues, and therefore, more receptive to this curriculum than the general population of residents.”  Comment: The students who elected to take the course early may have been more motivated to learn and master the course material. |

**Journal Article:**

Wundrich M, Schwartz C, Feige B, Lemper D, Nissen C, Voderholzer U. Empathy training in medical students - a randomized controlled trial. *Med Teach.* 2017;39(10):1096-1098.

| **Entry** | **Judgement** | **Support for judgement** |
| --- | --- | --- |
| Random sequence generation (selection bias) | Unclear risk | Quote: “A total of 158 3rd year medical students... were assigned into an intervention group… and a control group.”  Comment: Does not elucidate how assignments were made, how many students were in each group or demographic information of either group |
| Allocation concealment (selection bias) | Unclear risk | Quote: “A total of 158 3rd year medical students... were assigned into an intervention group… and a control group.”  Comment: Does not elaborate on how assignments were made or if students were aware of participation in study |
| Blinding of participants and personnel (performance bias) | High risk | Quote: “The empathy skills training consisted of an introduction course on empathy and an empathy skills training.”  Comment: It would be impossible to blind a student from whether or not they were participating in training focusing on empathy. |
| Blinding of outcome assessment (detection bias) - total empathy score | Low risk | Quote: “Empathy was rated in both groups by blinded experts and SPs.”  Comment: Experts & SPs blinded to intervention vs control group. |
| Blinding of outcome assessment (detection bias) - JSPE | High risk | Quote: “As a self-assessment, students filled out the… JSPE.”  Comment: Unable to blind this outcome measure because they are self reports. |
| Incomplete outcome data addressed (attrition bias) | Unclear risk | Quote: “The OSCE took place at the end of the three-week course.”  Comment: No report of number of students in intervention or control groups or if they were all present for the OSCE at the end of their respective courses. |
| Selective reporting (reporting bias) | Unclear risk | Comment: Study protocol not available. |
| Other Bias:  (Hawthorne effect) | Unclear risk | Quote: “The OSCE took place at the end of the three-week course.”  Comment: Unknown if students were aware that they were participating in a study but given the course was over 3 weeks, students could potentially discuss their experiences and how their training differed, cueing them into participation in a study, and a possible change in performance as a result. |
| Other Bias:  (other) | Unclear risk | Quote: “Potential measurement errors (e.g. biased ratings for likeable/congenial students) were discussed in the training.”  Comment: Unclear how this was discussed or whether this discussion would have had any subsequent impact on scoring. |

**Journal Article:**

Schweller M, Ribeiro DL, Celeri EV, de Carvalho-Filho MA. Nurturing virtues of the medical profession: does it enhance medical students' empathy? *Int J Med Educ.* 2017;8:262-267.

| **Entry** | **Judgement** | **Support for judgement** |
| --- | --- | --- |
| Random sequence generation (selection bias) | High risk | Quote: “Our study did not have a control group, narrowing the interpretation of the results.”  Comment: No randomization - self control before/after. |
| Allocation concealment (selection bias) | High risk | Quote: “This is a pretest-posttest study designed to assess the empathy levels of first-year medical students (n=166) comprising two consecutive classes (2012 and 2013) of a medical school in Brazil, performed before and after they participated in a curricular course called Health and Medicine (H&M).”  Comment: No randomization - self control. |
| Blinding of participants and personnel (performance bias) | High risk | Quote: “On the first day of class, we… shared with them the purpose of the H&M Course. Subsequently, we presented this study...”  Comment: Unable to blind - all participants aware of intervention. |
| Blinding of outcome assessment (detection bias) | High risk | Quote: “On the first day of class... the participants filled out the pretest of the student version of the Jefferson Scale of Physician Empathy (JSPE).” (and) “On the last day of class, they filled out the posttest of the JSPE (four months after the pretest application).”  Comment: Unable to blind - all participants aware of outcome assessment (self report). |
| Incomplete outcome data addressed (attrition bias) | Low risk | Quote: “This is a pretest-posttest study...of first-year medical students (n=166)... performed before and after they participated in a curricular course called Health and Medicine (H&M).”  Comment: No attrition. |
| Selective reporting (reporting bias) | Unclear risk | Comment: Protocol not available. |
| Other Bias:  (social desirability bias) | High risk | Comment: Potential for learners to want to answer questions in a way that would be viewed favorably by others. |
| Other Bias:  (maturation bias) | High risk | Quote: “The observed increase in the empathy levels of our first-year medical students may have been overestimated due to the maturation bias (a natural process that leads participants to change over time).”  Comment: This may have lead to higher post test scores. |
| Other Bias:  (Hawthorne effect) | High risk | Quote: “The observed increase in the empathy levels of our first-year medical students may have been overestimated due to...the Hawthorne effect (the tendency for people to perform better when participating in an experiment and being observed).”  Comment: This may have lead to higher post test scores. |

**Journal Article:**

LoSasso AA, Lamberton CE, Sammon M, et al. Enhancing Student Empathetic Engagement, History-Taking, and Communication Skills During Electronic Medical Record Use in Patient Care. *Acad Med.* 2017;92(7):1022-1027.

| **Entry** | **Judgement** | **Support for judgement** |
| --- | --- | --- |
| Random sequence generation (selection bias) | Unclear risk | Quote: “Participants in each six-week clerkship block were randomly assigned to the intervention group (n = 38) or to the control group (n = 32).”  Comment: While students were randomized, the method for this was not revealed. |
| Allocation concealment (selection bias) | High risk | Quote: “In consenting for the study, students in both groups were made aware that the study examined how the training may improve empathy, which could have led to some bias.” (and) “The intervention group underwent an additional one-hour training session on EMR-specific communication skills on their first day of orientation.”  Comment: It would be easy for students to determine which group they were allocated to. |
| Blinding of participants and personnel (performance bias) | High risk | Quote: “In consenting for the study, students in both groups were made aware that the study examined how the training may improve empathy, which could have led to some bias.” (and) “The intervention group underwent an additional one-hour training session on EMR-specific communication skills on their first day of orientation.”  Comment: It would be impossible to blind a student from whether or not they were participating in training focusing on empathy. |
| Blinding of outcome assessment (detection bias) - JSPPPE by SPs & faculty raters | Low risk | Quote: “The SP and faculty raters were blinded to whether students were in the intervention or control group.”  Comment: Blinding of assessors occurred. |
| Blinding of outcome assessment (detection bias) - JSE | High risk | Quote: “All participating students completed the JSE twice: once at the beginning of the six-week block … and again at the end of the block after the SP encounter.”  Comment: Unable to blind - all participants aware of this outcome assessment (self report). |
| Incomplete outcome data addressed (attrition bias) | Low risk | Quote: “Participants in each six-week clerkship block were randomly assigned to the intervention group (n = 38) or to the control group (n = 32).”  Comment: All participants were included in analysis in Table 1. |
| Selective reporting (reporting bias) | Unclear risk | Comment: Study protocol not available. |
| Other Bias:  (Hawthorne effect) | High risk | Quote: ““In consenting for the study, students in both groups were made aware that the study examined how the training may improve empathy, which could have led to some bias.”  Comment: Potential for change in behavior when aware of participation in a study. |
| Other Bias:  (other) | High risk | Quote: “Because the intervention training session did include participating in role-plays, it is possible that the role-plays alone could have bolstered communication and empathy skills and may account for differences between groups.”  Comment: Role-play in intervention only group could be confounder |

**Journal Article:**

Ruiz-Moral R, Perula de Torres L, Monge D, Garcia Leonardo C, Caballero F. Teaching medical students to express empathy by exploring patient emotions and experiences in standardized medical encounters. *Patient Educ Couns.* 2017;100(9):1694-1700.

| **Entry** | **Judgement** | **Support for judgement** |
| --- | --- | --- |
| Random sequence generation (selection bias) | High risk | Quote: “The course is part of the mandatory training… for 3rd-year medical students.”  Comment: No randomization - self control before/after. |
| Allocation concealment (selection bias) | High risk | Quote: “The course is part of the mandatory training… for 3rd-year medical students.”  Comment: No randomization - self control before/after. |
| Blinding of participants and personnel (performance bias) | High risk | Quote: “The study design, not blinded and without a control group, does not allow us to establish causal relationships...”  Comment: Impossible to blind participants from mandatory training, though it is unclear if students were aware that they were being studied. |
| Blinding of outcome assessment (detection bias) | Unclear risk | Quote: “The course was evaluated measuring the following: an external observer scored all students’ videotaped interviews” (and) “After the encounter, each SP immediately scored three aspects…”  Comment: It is unclear who the external observers were or if they were blinded. It is also unclear if the SPs were the same SPs throughout each of the student’s encounters, if they were blinded to which number encounter the student was participating in, or if they were even aware of the study. |
| Incomplete outcome data addressed (attrition bias) | Low risk | Quote: “The number of students enrolled in the course was 115.” (and) “All students carried out the first interview and 113 roleplayed the second.”  Comment: Low rate of attrition. |
| Selective reporting (reporting bias) | Unclear risk | Comment: Study protocol not available. |
| Other Bias:  (maturation bias) | High risk | Quote: “During six weeks students are deeply involved with patients in clinical encounters in hospital and primary care.”  Comment: It is unclear at what point in the 3rd year this course was performed. If performed in the beginning of the year where there may be a steep learning curve, there is a potential for improvement regardless of course involvement. Also no control arm for comparison. |
| Other Bias:  (Hawthorne effect) | Unclear risk | Comment: While participants would be aware of participation in mandatory training, it is unclear if students were aware that they were being studied. |

**Journal Article:**

Buffel du Vaure C, Lemogne C, Bunge L, et al. Promoting empathy among medical students: A two-site randomized controlled study. *J Psychosom Res.* 2017;103:102-107.

| **Entry** | **Judgement** | **Support for judgement** |
| --- | --- | --- |
| Random sequence generation (selection bias) | Low risk | Quote: “Students from Paris Diderot… were randomized… using computer-generated random numbers.” (and) “Among students from Paris Descartes, we took advantage of the randomization routinely performed each year by the University staff to allocate each student to one of three groups…”  Comment: Randomization with no significant difference in participants’ characteristics at baseline (Table 1). |
| Allocation concealment (selection bias) | Low risk | Quote: “Students from Paris Diderot… were randomized… using computer-generated random numbers.” (and) “Among students from Paris Descartes, we took advantage of the randomization routinely performed each year by the University staff to allocate each student to one of three groups…”  Comment: Allocation sequence could not be foreseen in advance of enrollment. |
| Blinding of participants and personnel (performance bias) | High risk | Quote: “Students and facilitators were aware of the allocated group”  Comment: It is impossible to blind participation in Balint group. |
| Blinding of outcome assessment (detection bias) - JSPE | High risk | Comment: Unable to blind - all participants aware of this outcome assessment (self report). |
| Blinding of outcome assessment (detection bias) - CARE | Low risk | Quote: “Standardized patients, OSCE’s observers and data analysts were kept blinded to the allocation.”  Comment: All outcome assessors were blinded. |
| Incomplete outcome data addressed (attrition bias) - JSPE | Low risk | Quote: “There was no significant difference regarding participants’ characteristics and group allocation between these 299 participants and the 53 students who did not complete the empathy assessment.” (and) “We included the 53 students who did not complete the empathy assessment, using their JSPE-MS score at baseline as the… score at follow-up. This sensitivity analysis yielded similar results.”  Comment: While there was a significant amount of students lost to follow up, it does not appear that there were significant differences between groups and inclusion of their data in sensitivity analysis did not change results at least for JSPE-MS score. |
| Incomplete outcome data addressed (attrition bias) - CARE | Unclear risk | Quote: “There was no significant difference regarding participants’ characteristics and group allocation between these 299 participants and the 53 students who did not complete the empathy assessment.”  Comment: It does not appear that there were significant differences between groups but it doesn’t appear that sensitivity analyses were performed for CARE measures so it is unclear what effect this could have had. |
| Selective reporting (reporting bias) | Low risk | Quote: “The protocol is registered at ClinicalTrials.gov (NCT02681380).” At ClinicalTrials.gov, “Primary Outcome Measures: 1. Care… Scale to measure empathy by a simulated patient, during a simulated visit.”  Comment: The original primary outcome measure was reported in addition to another outcome measure of JSPE-MS pre-/post- participation. |
| Other Bias:  (social desirability bias) | High risk | Quote: “Several explanations could account for the effects observed… it could result from increased social desirability of empathy rather than from increased empathic abilities per se.”  Comment: Potential for learners to want to answer questions in a way that would be viewed favorably by others. |
| Other Bias:  (Hawthorne effect) | High risk | Quote: “Students and facilitators were aware of the allocated group”  Comment: Awareness of participation in a study could alter behavior. |

**Journal Article:**

Zazulak J, Sanaee M, Frolic A, et al. The art of medicine: arts-based training in observation and mindfulness for fostering the empathic response in medical residents. *Med Humanit.* 2017;43(3):192-198.

| **Entry** | **Judgement** | **Support for judgement** |
| --- | --- | --- |
| Random sequence generation (selection bias) | High risk | Quote: “Fifteen residents… volunteered to participate (ie, self-selected).” (and) “Twenty...residents acted as controls.”  Comment: Prospective cohort study. No randomization. |
| Allocation concealment (selection bias) | High risk | Quote: “Fifteen residents… volunteered to participate (ie, self-selected).”  Comment: Residents self-selected - no allocation to conceal. |
| Blinding of participants and personnel (performance bias) | High risk | Quote: “Fifteen residents… volunteered to participate (ie, self-selected).”  Comment: Residents self-selected therefore they are aware of intervention versus control participation. |
| Blinding of outcome assessment (detection bias) | High risk | Quote: “All participants completed three validated psychometric scales.”  Comment: Self reported scales therefore no way to blind outcome assessment. |
| Incomplete outcome data addressed (attrition bias) | Low risk | Quote: “All participants completed three validated psychometric scales on the first and last days of the programme.”  Comment: No attrition. |
| Selective reporting (reporting bias) | Unclear risk | Comment: Study protocol not available. |
| Other Bias:  (social desirability bias) | High risk | Comment: Potential for learners to want to answer questions in a way that would be viewed favorably by others. |
| Other Bias:  (Hawthorne effect) | Unclear risk | Comment: The tendency to perform better when participating in an experiment may have lead to higher post test scores, though given no difference in the IRI or compassion subscale this bias was likely minimal |
| Other Bias:  (small sample size) | Unclear risk | Quote: “Fifteen residents… volunteered to participate (ie, self-selected).” (and) “Twenty...residents acted as controls.”  Comment: Relatively small sample size may have limited the ability to detect a difference in IRI or compassion scale. |
| Other Bias  (other) | High risk | Quote: “Fifteen residents… volunteered to participate (ie, self-selected).” (and) “Twenty...residents acted as controls.”  Comment: May be inherent differences in individuals who self-selected to participate - may be more motivated to improve their compassion skills. |

**Journal Article:**

Delacruz N, Reed S, Splinter A, et al. Take the HEAT: A pilot study on improving communication with angry families. *Patient Educ Couns.* 2017;100(6):1235-1239.

| **Entry** | **Judgement** | **Support for judgement** |
| --- | --- | --- |
| Random sequence generation (selection bias) | High risk | Quote: “All first-year pediatrics and internal medicine-pediatrics residents… were invited to participate.” (and) “Resident baseline communication skills were assessed via simulated encounter… Post-workshop skills were assessed with another simulated encounter.”  Comment: No control group - before/after self control. |
| Allocation concealment (selection bias) | High risk | Quote: “All first-year pediatrics and internal medicine-pediatrics residents… were invited to participate.” (and) “Resident baseline communication skills were assessed via simulated encounter… Post-workshop skills were assessed with another simulated encounter.”  Comment: No randomization - self control. |
| Blinding of participants and personnel (performance bias) | High risk | Quote: “Resident baseline communication skills were assessed via simulated encounter and the education was delivered at a resident retreat. Post-workshop skills were assessed with another simulated encounter.”  Comment: Unable to blind - self control. |
| Blinding of outcome assessment (detection bias) | Unclear risk | Quote: “Five raters assessed pre-workshop SP encounters using the novel evaluation tool… Based on...degree of agreement… it was determined that scores from one singler rater, rater 1, could justifiably be used for both pre- and post-workshop encounters.”  Comment: Does not specify who raters are or whether or not they were blinded. |
| Incomplete outcome data addressed (attrition bias) | Unclear risk | Quote: “33 of 47 residents participated in all phases of the study. Clinical responsibilities prevented some residents from participating.”  Comment: Large number of residents not included secondary to clinical responsibilities - unknown if this could have lead to difference in outcome. |
| Selective reporting (reporting bias) | Unclear risk | Comment: Study protocol not available. |
| Other Bias:  (maturation bias) | High risk | Comment: Natural change/improvement in time may have lead to higher post test scores. No control group to compare. |
| Other Bias:  (Hawthorne effect) | High risk | Comment: The tendency to perform better when participating in an experiment may have lead to higher post test scores. |
| Other Bias:  (small sample size) | Unclear risk | Comment: Relatively small sample size may have increased the chances that results were due to chance and may also limit generalizability. |
| Other Bias:  (other) | Unclear risk | Quote: “All first-year pediatrics and internal medicine-pediatrics residents… were invited to participate.” (and) “33 of 47 residents participated in all phases of the study. Clinical responsibilities prevented some residents from participating.”  Comment: Unknown if clinical responsibilities were the only reason for non-participation. May also be inherent differences in individuals who self-selected to participate - may be more motivated to improve their compassion skills. |

**Journal Article:**

Flint H, Meyer M, Hossain M, Klein M. Discussing Serious News. *American Journal of Hospice & Palliative Medicine.* 2017;34(3):254-257.

| **Entry** | **Judgement** | **Support for judgement** |
| --- | --- | --- |
| Random sequence generation (selection bias) | High risk | Quote: “The residents who participated were a representative sample of the pediatric residency program.”  Comment: No randomization - before/after self control. |
| Allocation concealment (selection bias) | High risk | Quote: “The residents who participated were a representative sample of the pediatric residency program.”  Comment: No randomization - before/after self control. |
| Blinding of participants and personnel (performance bias) | High risk | Quote: “Pediatric residents participated in a 3-hour communication skills workshop during a required advocacy rotation.”  Comment: Impossible to blind residents to participation. |
| Blinding of outcome assessment (detection bias) | Unclear risk | Comment: Unable to blind a self reported outcome assessment |
| Incomplete outcome data addressed (attrition bias) | Low risk | Quote: “Forty-two (100%) participants completed the survey immediately following participation.”  Comment: No attrition. |
| Selective reporting (reporting bias) | Unclear risk | Comment: Study protocol not available. |
| Other Bias:  (social desirability bias) | High risk | Comment: Potential for learners to want to answer questions in a way that would be viewed favorably by others. |
| Other Bias:  (Hawthorne effect) | Unclear risk | Comment: While all residents were aware of participation in the workshop, it is unclear if they were aware that they were part of a study. There could be the potential to improve performance when aware of participation in a study. |
| Other Bias:  (small sample size) | Unclear risk | Comment: Relatively small sample size may have increased the chances that results were due to chance and may also limit generalizability. |

**Journal Article:**

Ditton-Phare P, Sandhu H, Kelly B, Kissane D, Loughland C. Pilot Evaluation of a Communication Skills Training Program for Psychiatry Residents Using Standardized Patient Assessment. *Acad Psychiatry.* 2016;40(5):768-775.

| **Entry** | **Judgement** | **Support for judgement** |
| --- | --- | --- |
| Random sequence generation (selection bias) | High risk | Quote: “A number of limitations associated with this study, most notably the use of a pre/post design without a control group.”  Comment: Not randomized - self control & a “quasi-control” group (5 trainees that did not attend training but completed pre- and post-SPA). |
| Allocation concealment (selection bias) | High risk | Quote: “Thirty trainees... undertaking their vocational specialty in psychiatry participated in… SPAs...pre- and post-ComPsych training.”  Comment: Not randomized - pre/post self control. |
| Blinding of participants and personnel (performance bias) | High risk | Quote: “Thirty trainees... undertaking their vocational specialty in psychiatry participated in… SPAs...pre- and post-ComPsych training.”  Comment: Unable to blind participation in ComPsych training or digital recording of SPAs. |
| Blinding of outcome assessment (detection bias) | Low risk | Quote: “Coders were blind to whether each recording was pre- or post-training and were not informed of the study methodology.”  Comment: Expert coders independent of ComPsych team. |
| Incomplete outcome data addressed (attrition bias) | Low risk | Quote: “Two expert coders... were employed and trained to view 60 digitally recorded SPAs.”  Comment: All trainees included in assessment (30 trainees, each with 1 pre- and one post-SPA). |
| Selective reporting (reporting bias) | Unclear risk | Comment: No study protocol available. |
| Other bias:  (Hawthorne effect) | Unclear risk | Comment: While all participants were aware of the naturally occuring education program, it is unclear if they were aware of participation in a study. If so, this could alter their behavior. |
| Other bias:  (small sample size) | Unclear risk | Comment: There was a non-statistically significant improvement in empathetic communication. The relatively small sample size may have limited the ability to detect a statistically significant difference in empathetic communication. |
| Other Bias:  (other) | Unclear risk | Quote: “Confounding the findings of our study was differences in elapsed time between ComPysch training and post-SPA delivery (which ranged from 4 weeks at the first data collection to the same day at the final data collection) may have impacted on the facility of the SPA to gauge the immediate impact of the training in the first and second data collections.”  Comment: There is a potential for maturation bias over the 4 weeks for individuals in the first data collection. Conversely, a 4 week time lag could also lead to a decline of memory retention and decrease the chances of picking up a significant difference compared to those whose data collection would have been on the same day as training. |
| Other bias:  (other) | Unclear risk | Quote: “Trainees participated in either one or both modules… depending on which training date they attended (across the four possible data collection points). This was not randomly allocated.” (and) “Significant increases were seen for those who had higher dose of training… These findings need to be tempered, however, by the fact that those who attended two sessions were possibly more interested in and committed to the training, and were perhaps most likely to modify their behavior.” (and) “While stated reasons for attrition in the level 2 evaluation were mostly illness or work commitments, it is also possible that participants harbored anxiety about being filmed (as was overtly the case with one participant).” (and) “Furthermore, the group not trained may be a subset of trainees who most need training (e.g., they may lack confidence and do not wish to be scrutinized).”  Comment: There is the potential for significant baseline differences in those who participated in training and those who decided to participate in more than one module. |

**Journal Article:**

Boissy A, Windover AK, Bokar D, et al. Communication Skills Training for Physicians Improves Patient Satisfaction. *J Gen Intern Med.* 2016;31(7):755-761.

| **Entry** | **Judgement** | **Support for judgement** |
| --- | --- | --- |
| Random sequence generation (selection bias) | High risk | Quote: “All… physicians… who were mandated to attend an 8-h...experiential communication skills training between 1 August 2013 and 30 April 2014.” (and) “Physicians who participated in an earlier version or who had not yet taken the course were included as controls.”  Comment: No randomization - matched cohorts. |
| Allocation concealment (selection bias) | Unclear risk | Quote: “All… physicians… who were mandated to attend an 8-h...experiential communication skills training between 1 August 2013 and 30 April 2014.” (and) “Physicians who participated in an earlier version or who had not yet taken the course were included as controls.”  Comment: Allocation determined by dates as above which participants could potentially know. |
| Blinding of participants and personnel (performance bias) | Unclear risk | Quote: “Data was entered into a registry approved by the Cleveland Clinic Institutional Review Board. All participants had the option to exclude their data from the registry. The study, which used existing data, was deemed exempt.”  Comment: Participants would know whether or not they participated in the training though it is unclear if they were aware that their subsequent HCAHPS, CGCAHPS & survey responses would be included as part of a study. It seems that they would know that data was being collected if they had the option to exclude their data from the registry, however, it’s unclear if they knew how the data was being used. |
| Blinding of outcome assessment (detection bias) - CGCAHPS & HCAHPS scores | Unclear risk | Quote: “We then collected HCAHPS and CGCAHPS scores for physician communication for 6 months before and 6 months after the course date (or corresponding pseudo course date) using their… NPI numbers.”  Comment: It is unclear if assessors were blinded to physician allocation during data assessment. |
| Blinding of outcome assessment (detection bias) - JSPE | High risk | Quote: “Physicians were asked to complete pre- and post-course surveys on the day of training and at 3 months post-course.”  Comment: Participants cannot be blinded to self-reported questionnaire outcome assessment. |
| Incomplete outcome data addressed (attrition bias) | High risk | Quote: “NPI-matched follow-up scores were available at 3 months for 16% of the physicians.” (and) “Physicians were excluded if they did not have… at least five pre- and five post-HCAHPS… or… CGCAHPS scores.”  Comment: Per figure 1, intervention group went from 1537 to 143 for JSE (low response rate), 204 for HCAHPS, 443 for CGCAHPS; control from 1951 to 230 for HCAHPS, 478 for CGCAHPS. |
| Selective reporting (reporting bias) | Unclear risk | Comment: Study protocol not available. |
| Other Bias:  (social desirability bias) | Unclear risk | Quote: “We included some self-reported outcomes, and reporting was not anonymous.”  Comment: Seems less likely given participants were not hesitant to express skepticism about course initially, likely minimizing social desirability bias in the JSPE scores. |
| Other Bias:  (chance bias) | Unclear risk | Quote: “For CGCAHPS & HCAHPS groups, intervention physicians had more years in practice and were more likely to be male.”  Comment: Baseline difference in groups could be a confounder. |
| Other Bias:  (other) | High risk | Quote: “Due to its observational nature, we could not rule out other causes for improvement in scores…”  Comment: Attempted to control this with use of controls but controls were participants at other predetermined time sets - other institutional factors during these times could have an effect. |

**Journal Article:**

Foster A, Chaudhary N, Kim T, et al. Using Virtual Patients to Teach Empathy: A Randomized Controlled Study to Enhance Medical Students' Empathic Communication. *Simul Healthc.* 2016;11(3):181-189.

| **Entry** | **Judgement** | **Support for judgement** |
| --- | --- | --- |
| Random sequence generation (selection bias) | Unclear risk | Quote: “First-year medical students were randomly assigned to one of the following distinct groups…”  Comment: Random assignments though unclear method for randomization. |
| Allocation concealment (selection bias) | Unclear risk | Quote: “First-year medical students were randomly assigned to one of the following distinct groups…”  Comment: Method to generate allocations not known. |
| Blinding of participants and personnel (performance bias) | High risk | Quote: “The VP scenario content was identical for all study groups… The scenario was technically enhanced with empathy feedback or a backstory for the respective intervention groups.”  Comment: It would be impossible to blind participants to whether or not they received empathy feedback or viewed a video backstory for their VP encounters. |
| Blinding of outcome assessment (detection bias) | Low risk | Quote: “Each live SP-student interaction was videotaped and transcribed by a study investigator.” (and) “The SPs were blinded to the students’ study group assignment.” (and) “Measures were taken to label the transcripts in each student… such that the source of the transcripts was not identifiable to the assessors.”  Comment: SPs and trained assessors were blinded to intervention vs control group. |
| Incomplete outcome data addressed (attrition bias) | Low risk | Quote: “All students completed all elements of the study.”  Comment: No attrition. |
| Selective reporting (reporting bias) | Unclear risk | Comment: Study protocol not available. |
| Other bias:  (Hawthorne effect) | Unclear risk | Quote: “Recruitment was sought... “ (and) “Informed consent was obtained…”  Comment: Students were fully aware of participation in a study which could potentially alter their behavior, though this would likely be seen equally across all groups. |
| Other Bias:  (sampling bias) | High risk | Quote: “Recruitment was sought… the students were contacted through mass emails, flyers, or privated word of mouth.”  Comment: There may be baseline differences in students who selected to participate in the study vs those who did not. |

**Journal Article:**

Orloski CJ, Tabakin ER, Myers JS, Shofer FS, Mills AM. Grab a SEAT: Sit, engage, ask, teach an emergency department performance improvement initiative. *Ann Emerg Med.* 2016;68:4 Supplement 1 (S130-)

| **Entry** | **Judgement** | **Support for judgement** |
| --- | --- | --- |
| Random sequence generation (selection bias) | High risk | Quote: “This is an ongoing before-after study.”  Comment: Prospective cohort - no randomization. |
| Allocation concealment (selection bias) | High risk | Quote: “This is an ongoing before-after study.”  Comment: Prospective cohort - no randomization to conceal. |
| Blinding of participants and personnel (performance bias) | High risk | Quote: “At one ED, patients and providers received the intervention while the second ED served as the control group.”  Comment: Participants could know to which group they are assigned based on which ED they work in. |
| Blinding of outcome assessment (detection bias) | Unclear risk | Comment: Abstract only - details of methods not mentioned. |
| Incomplete outcome data addressed (attrition bias) | Unclear risk | Comment: Abstract only - details of methods/results not mentioned. |
| Selective reporting (reporting bias) | Unclear risk | Comment: Study protocol not available. |
| Other bias:  (Hawthorne effect) | Unclear risk | Comment: While participants would be aware of educational outreach, it is unclear if they were aware of participation in a study which could potentially alter their behavior at the beside. |

**Journal Article:**

Duke P, Grosseman S, Novack DH, Rosenzweig S. Preserving third year medical students' empathy and enhancing self-reflection using small group "virtual hangout" technology. *Med Teach.* 2015;37(6):566-571.

| **Entry** | **Judgement** | **Support for judgement** |
| --- | --- | --- |
| Random sequence generation (selection bias) | High risk | Quote: “All 259 third year students enrolled at the beginning of the 2012 school year… were invited to participate.”  Comment: No randomization - self control before/after. |
| Allocation concealment (selection bias) | High risk | Quote: “All 259 third year students enrolled at the beginning of the 2012 school year… were invited to participate.”  Comment: No randomization - before/after study. |
| Blinding of participants and personnel (performance bias) | High risk | Quote: “Those who agreed to participate signed informed consent forms and completed the questionnaires.”  Comment: Unable to blind students to participation. |
| Blinding of outcome assessment (detection bias) | High risk | Quote: “Those who agreed to participate signed informed consent forms and completed the questionnaires.”  Comment: Unable to blind students to method of assessment with a self-reported questinonaire. |
| Incomplete outcome data addressed (attrition bias) | High risk | Quote: “Post-course questionnaires (Time 2) were administered by the faculty facilitator of each group, but some failed to either distribute or collect the questionnaires, resulting in data loss.”  Comment: No Time 2 questionnaires in almost 50% (Table 1). |
| Selective reporting (reporting bias) | Unclear risk | Comment: Study protocol not available. |
| Other Bias:  (social desirability bias) | High risk | Quote: “Also, in any self-reported survey, social desirability bias is possible.”  Comment: May be tempered by non-penalizing testing situation and anonymity (disinterested staff person recorded their information). |
| Other Bias:  (maturation bias) | High risk | Comment: Natural change/improvement in time may have lead to higher post test scores. No control group to compare. |
| Other Bias:  (Hawthorne effect) | High risk | Comment: The tendency to perform better when participating in an experiment may have lead to higher post test scores. |
| Other Bias:  (other) | Unclear risk | Quote: “Because we did not have a control group, we do not know whether preservation of empathy was related to this particular course or to other institutional changes or variables.”  Comment: Potential institutional confounders. |

**Journal Article:**

Lusilla-Palacios P, Castellano-Tejedor C. Training a Spinal Cord Injury Rehabilitation Team in Motivational Interviewing. *Rehabil Res Pract.* 2015;2015:358151.

| **Entry** | **Judgement** | **Support for judgement** |
| --- | --- | --- |
| Random sequence generation (selection bias) | High risk | Quote: “The whole rehabilitation staff… was invited to participate in the study (N=63) on a voluntary basis.”  Comment: No randomization - before/after self control. |
| Allocation concealment (selection bias) | High risk | Quote: “The whole rehabilitation staff… was invited to participate in the study (N=63) on a voluntary basis.”  Comment: No randomization - before/after self control. |
| Blinding of participants and personnel (performance bias) | High risk | Quote: “The whole rehabilitation staff… was invited to participate in the study (N=63) on a voluntary basis.”  Comment: No blinding - all participants aware of participation. |
| Blinding of outcome assessment (detection bias) | High risk | Quote: “In these sessions, carried out by the main researcher of the study…”  Comment: Unable to blind participants from self-reported outcome assessment (JSPE). |
| Incomplete outcome data addressed (attrition bias) | High risk | Quote: “From the initial pool of 63 professionals… a total of 45 professionals were assessed (before/after).” (and) “Missing sample in post training assessments was due to retirement (n=1), sick or maternity leaves (n=5), change of service (n=7) or not wanting to answer again the questionnaires (n=5).”  Comment: Only 71.4% of a relatively small sample had post-training assessments. |
| Selective reporting (reporting bias) | High risk | Quote: “The full protocol of this study is registered in ClinicalTrials.gov (identifier: NCT01889940).”  Comment: At ClinicalTrials.gov, the primary outcome measure is listed as the Picker Patient Experience Questionnaire (PPE-33). There are also 6 secondary outcome measures followed by 6 “other” outcome measures. Ultimate outcomes reported in the paper (JSPE & MBI) were listed under “other” outcome measures. |
| Other Bias:  (social desirability bias) | Unclear risk | Quote: “Prior to the training, the staff working in the SCI unit displayed a high average of almost 19 years working in this specific field.”  Comment: There is a possibility that the learners could have self reported the JSPEs as higher before the intervention because of self expectation of a high degree of empathy with a large number of years in the field. This could potentially lead to a ceiling effect and inability to detect a difference post-intervention. |
| Other Bias:  (maturation bias) | Unclear risk | Quote: “It was a three year protocol.” (and) “Prior to the training, the staff working in the SCI unit displayed a high average of almost 19 years working in this specific field.”  Comment: There is the potential for natural improvement of empathy with time and this training did occur over a large period of time. However, this may be minimized by the fact that the participants as a whole were already very experienced in their field with less of a chance of change over time (versus those early in their careers who have the potential for a steep learning curve). |
| Other Bias:  (Hawthorne effect) | Unclear risk | Quote: “The whole rehabilitation staff… was invited to participate in the study (N=63) on a voluntary basis.”  Comment: Staff were aware of participation in a study which could potentially alter performance, though would have expected a change in the JSPE if this were a significant confounder. |
| Other Bias:  (other) | Unclear risk | Quote: “It was a three year protocol.”  Comment: There could be many institutional confounders over this three year period which could have affected change in empathy. |

**Journal Article:**

Potash JS, Chen JY, Lam CL, Chau VT. Art-making in a family medicine clerkship: how does it affect medical student empathy? *BMC Med Educ.* 2014;14:247.

| **Entry** | **Judgement** | **Support for judgement** |
| --- | --- | --- |
| Random sequence generation (selection bias) | High risk | Quote: “Students were randomly assigned into either an arts-making or a clinical problem-solving workshop according to their schedule during the FM curriculum.”  Comment: Assigning students by schedule is non-random. |
| Allocation concealment (selection bias) | High risk | Quote: “Students were randomly assigned… according to their schedule during the FM curriculum.”  Comment: Participants could foresee assignment based on their schedule. |
| Blinding of participants and personnel (performance bias) | High risk | Quote: “Students in the arts-making workshop were led in a guided visualization...Afterwards, they recorded colors, smells, sounds, objects and feelings… Next they created a drawing or painting… ” (and) “Students in the clinical problem-solving workshop observed or participated in a role play with one of the facilitators…”  Comment: Participants would be fully aware which group they are participating in. |
| Blinding of outcome assessment (detection bias) | High risk | Quote: “Potential researcher bias and subjectivity were minimized by independent coding.”  Comment: W data analysis seems to have been performed by individuals independent of the researchers, the outcome assessment was still a self-report measure and therefore participants would not be blinded to this. |
| Incomplete outcome data addressed (attrition bias) | High risk | Quote: “Of the 161 eligible participants, 152 consented to take part in the study and 106 completed both pre-and post-questionnaire measures and submitted all assignments for a participation rate of 70%.”  Comment: High attrition rate - unknown if difference in those who did versus did not complete the post-questionnaire. |
| Selective reporting (reporting bias) | Unclear risk | Comment: Study protocol not available. |
| Other Bias:  (social desirability bias) | Unclear risk | Comment: Potential for students to respond in a socially desirable way, though if this were the case, a larger change in JSE would be expected. |
| Other Bias:  (maturation bias) | Uncear risk | Quote: “It is difficult to determine if changes are a result of the workshops or due to other experiences encountered over the course of the rotation.”  Comment: There may have been a natural change/improvement over time with pre-test performed at the beginning of the FM block and post-tests at the end of the FM block. However, if this were the case, a statistically significant change in JSE would be expected. |
| Other Bias:  (Hawthorne effect) | Unclear risk | Comment: Potential for students to improve performance when they are aware that they are participating in a study, though if this were the case, a larger change in JSE would be expected. |

**Journal Article:**

Nasr Esfahani M, Behzadipour M, Jalali Nadoushan A, Shariat SV. A pilot randomized controlled trial on the effectiveness of inclusion of a distant learning component into empathy training. *Med J Islam Repub Iran.* 2014;28:65.

| **Entry** | **Judgement** | **Support for judgement** |
| --- | --- | --- |
| Random sequence generation (selection bias) | High risk | Quote: “Subjects of the study included 14 first year residents of psychiatry…” “Subjects were randomly allocated to two groups of 7 residents, considering gender as a stratum to have two male residents in each group.”  Comment: Do not specify method of randomization, though at least part of assignment was not random with regard to handling of gender. |
| Allocation concealment (selection bias) | Unclear risk | Quote: “Subjects were randomly allocated to two groups of 7 residents, considering gender as a stratum to have two male residents in each group.”  Comment: Method of randomization unknown, therefore unknown if it could be predicted by participants. |
| Blinding of participants and personnel (performance bias) | High risk | Quote: “One group...was planned to attend the two days of the workshop (attending group) and the other group to view the videotape of the first day and to receive a text on the topic of communication skills and to attend the second session of the workshop (distance learning group).”  Comment: Impossible to blind participants to attending vs distance learning group. |
| Blinding of outcome assessment (detection bias) - JSE | High risk | Comment: It is impossible to blind participants from filling out the JSE, a self-reported outcome measure. |
| Blinding of outcome assessment (detection bias) - JSPPPE & OAE | Low risk | Quote: “The raters and the SP were blind to the group status of the subjects.”  Comment: All assessors blinded. |
| Incomplete outcome data addressed (attrition bias) - JSE | Low risk | Quote: “All of the subjects completed the JSE before and three months after the intervention.”  Comment: No attrition. |
| Incomplete outcome data addressed (attrition bias) - JSPPPE & OAE | Unclear risk | Comment: It is not reported if all 14 participants had JSPPPE & OAE scores. |
| Selective reporting (reporting bias) | Low risk | Quote: “The study is registered as a clinical trial in the Iranian Registry for Clinical Trials website with the IRCT ID of IRCT2012090510749N1.”  Comment: No change from original study protocol. |
| Other Bias:  (social desirability bias) - JSE | Unclear risk | Comment: Potential for residents to respond in a socially desirable way, though if this were the case, a larger change in JSE would be expected. |
| Other Bias:  (maturation bias) | Unclear risk | Quote: “In the objective evaluation of the subjects 3 months after the intervention...”  Comment: There may have been a natural change/improvement in both groups over 3 months time. |
| Other Bias:  (Hawthorne effect) | Unclear risk | Quote: “All of the participants… signed a written form of informed consent.”  Comment: Potential for residents to improve performance when they are aware that they are participating in a study. |
| Other Bias:  (small sample size) | High risk | Comment: Only 14 participants - may not be powered enough to detect a change (for JSPE or JSPPPE) or alternatively could increase the possibility that detected difference is due to chance (OAE). |
| Other Bias:  (chance bias) | High risk | Quote: “The two groups did not have equal empathy levels before the intervention.”  Comment: While the difference did not reach statistical significance, it is possible that the group with higher empathy could have more motivation to learn about empathy. |

**Journal Article:**

Williams B, Sadasivan S, Kadirvelu A, Olaussen A. Empathy levels among first year Malaysian medical students: an observational study. *Adv Med Educ Pract.* 2014;5:149-156.

| **Entry** | **Judgement** | **Support for judgement** |
| --- | --- | --- |
| Random sequence generation (selection bias) | High risk | Quote: “The study used a before and after repeated-measures design.”  Comment: No randomization - before/after self control. |
| Allocation concealment (selection bias) | High risk | Quote: “The study used a before and after repeated-measures design.”  Comment: No randomization - before/after self control. |
| Blinding of participants and personnel (performance bias) | High risk | Quote: “A 2-hour interactive empathy workshop was held for first year medical students during their 3-day orientation to the medicine program.” (and) “Attendance...was compulsory for all students.”  Comment: Impossible to blind participants from participation in workshop. |
| Blinding of outcome assessment (detection bias) | High risk | Comment: It is impossible to blind students to the method of assessment (self survey). |
| Incomplete outcome data addressed (attrition bias) | Low risk | Quote: “A total of 122 first year medical students completed the before and after questionnaires, giving a response rate of 100%.”  Comment: No attrition. |
| Selective reporting (reporting bias) | Unclear risk | Comment: Study protocol not available. |
| Other Bias:  (social desirability bias) | High risk | Quote: “Self-reported measures have inherent respondent bias and in no way reflect what might occur in actual clinical practice.”  Comment: Tendency for participants to respond in a socially desirable way. |
| Other Bias:  (maturation bias) | High risk | Quote: “Whether the workshop was responsible for this increased level of empathy is difficult to determine.”  Comment: Five weeks between initial JSPE & post-empathy workshop JSPE during a time with a potential steep learning curve (first year of medical school) - improvement could be secondary to other experiences/other natural growth over those 5 weeks. |
| Other Bias:  (Hawthorne effect) | High risk | Quote: “A total of 122 students… consented to take part in the study.”  Comments: Awareness of participation in a study could lead to improved behavior/reporting. |

**Journal Article:**

Airagnes G, Consoli SM, De Morlhon O, Galliot AM, Lemogne C, Jaury P. Appropriate training based on Balint groups can improve the empathic abilities of medical students: a preliminary study. *J Psychosom Res.* 2014;76(5):426-429.

| **Entry** | **Judgement** | **Support for judgement** |
| --- | --- | --- |
| Random sequence generation (selection bias) | High risk | Quote: “Even if we cannot exclude a selection bias in the choice of optional certificates… or in the consent to participate in our study…”  Comment: No randomization - students self selected into intervention group. |
| Allocation concealment (selection bias) | High risk | Quote: “By the short title of ‘Balint group’ we will refer to all fourth-year medical students… who participated in the optional certificate ‘doctor-patient relationship training.”  Comment: No randomization - students self selected into intervention group therefore no allocation to conceal. |
| Blinding of participants and personnel (performance bias) | High risk | Quote: “The study was presented as a research on doctor-patient relationship.” (and) “The following questionnaires were filled in by all the participants before and after the end of their optional certificate: The French version of the IRI...”  Comment: Unable to blind participation in Balint group or method of evaluation. |
| Blinding of outcome assessment (detection bias) | High risk | Quote: “The following questionnaires were filled in by all the participants before and after the end of their optional certificate: The French version of the IRI...”  Comment: Unable to blind method of outcome assessment with use of a self-report measure. |
| Incomplete outcome data addressed (attrition bias) | Low risk | Quote: “Sixty participated in the certificate on patient-doctor relationship...34 of them (57%) accepted to participate in our student and filled out the questionnaires; and 379 participated in other optional certificates: 129 of them (34%) filled in the questionnaires.”  Comment: No attrition. |
| Selective reporting (reporting bias) | Unclear risk | Comment: Study protocol not available. |
| Other Bias:  (social desirability bias) | Unclear risk | Comment: Potential for students to respond in a socially desirable way, though if this were the case, a larger change in IRI would be expected. |
| Other Bias:  (maturation bias) | Unclear risk | Quote: “Before the training sessions and 4 months later, they filled up the IRI..”  Comment: There may have been a natural change/improvement in all groups over 4 months time. |
| Other Bias:  (Hawthorne effect) | Unclear risk | Quote: “The study was presented as a research on doctor-patient relationship.”  Comment: Potential for students to improve performance when they are aware that they are participating in a study. |
| Other Bias:  (chance bias) | Unclear risk | Quote: “There were fewer students who were currently undergoing a personal psychotherapy in the Balint group than in the control group. Women had higher IRI subscores than men, and Empathetic-Approach was higher in students with lower SES.”  Comment: Baseline differences between groups could bias outcomes. |
| Other Bias:  (selection bias) | Unclear risk | Quote: “Even if we cannot exclude a selection bias in the choice of optional certificates…”  Comment: Students who self selected into the optional “doctor-patient relationship training” likely have a higher interest in improving their empathic abilities. |

**Journal Article:**

Schweller M, Costa FO, Antonio MA, Amaral EM, de Carvalho-Filho MA. The impact of simulated medical consultations on the empathy levels of students at one medical school. *Acad Med.* 2014;89(4):632-637.

| **Entry** | **Judgement** | **Support for judgement** |
| --- | --- | --- |
| Random sequence generation (selection bias) | High risk | Quote: “All 123 students who were in their sixth year… participated in the intervention.” (and) “Participation in the activity was voluntary for students who were in their fourth year.”  Comment: No randomization - before/after self control. |
| Allocation concealment (selection bias) | High risk | Quote: “All 123 students who were in their sixth year… participated in the intervention.” (and) “Participation in the activity was voluntary for students who were in their fourth year.”  Comment: No randomization - before/after self control therefore no allocation to conceal. |
| Blinding of participants and personnel (performance bias) | High risk | Quote: “All 123 students who were in their sixth year… participated in the intervention.” (and) “Participation in the activity was voluntary for students who were in their fourth year.”  Comment: Impossible to blind participation in the activity (simulation with SPs). |
| Blinding of outcome assessment (detection bias) | High risk | Comment: It is impossible to blind participants from filling out the JSPE or IRI, both self-reported outcome measures. |
| Incomplete outcome data addressed (attrition bias) | Unclear risk | Comment: While number of participants involved in the intervention are reported, the number who performed pre & post JSPE & IRI were not reported. |
| Selective reporting (reporting bias) | Unclear risk | Quote: “Our research project was first registered with the National Committee for Ethics in Research.”  Comment: Unable to find research protocol. |
| Other Bias:  (social desirability bias) | High risk | Comment: Tendency for students to self report in a socially desirable way. |
| Other Bias:  (maturation bias) | High risk | Quote: “One limitation of our study is the possibility that our results were affected by the maturation bias.”  Comment: Natural process in which participants could have changed over the 30 day time period. |
| Other Bias:  (Hawthorne effect) | High risk | Quote: “One limitation of our study is the possibility that our results were affected by...the Hawthorne effect.”  Comment: Tendency for people to perform better when being observed. |

**Journal Article:**

Graham KL, Green S, Kurlan R, Pelosi JS. A patient-led educational program on Tourette Syndrome: impact and implications for patient-centered medical education. *Teach Learn Med.* 2014;26(1):34-39.

| **Entry** | **Judgement** | **Support for judgement** |
| --- | --- | --- |
| Random sequence generation (selection bias) | High risk | Quote: “The current study is a pretest-posttest design in which participants’ self-reported empathy was assessed before and after a 1-hour patient-led presentation.”  Comment: No randomization - before/after self control. |
| Allocation concealment (selection bias) | High risk | Quote: “The current study is a pretest-posttest design in which participants’ self-reported empathy was assessed before and after a 1-hour patient-led presentation.”  Comment: No randomization - before/after self control. |
| Blinding of participants and personnel (performance bias) | High risk | Quote: “The current study is a pretest-posttest design in which participants’ self-reported empathy was assessed before and after a 1-hour patient-led presentation.”  Comment: Unable to blind participants to participation in session. |
| Blinding of outcome assessment (detection bias) | High risk | Comment: It is impossible to blind participants in filling out the JSPE, a self-reported outcome measure. |
| Incomplete outcome data addressed (attrition bias) | Unclear risk | Quote: “Seventy-nine medical residents… participated in the study.”  Comment: It is not reported if all 79 residents filled out the pre and post tests. |
| Selective reporting (reporting bias) | Unclear risk | Comment: Study protocol not available. |
| Other Bias:  (social desirability bias) | Unclear risk | Quote: “Participants self-reported empathy was assessed.”  Comment: Tendency for participants to respond in a socially desirable way. |
| Other Bias:  (Hawthorne effect) | Unclear risk | Comment: While participants would be aware of participation in a patient-led presentation and filling out of the JSPE, it is unclear if they were aware they were participating in a study. If they were, this could lead to improved performance/reporting. |

**Journal Article:**

Bays AM, Engelberg RA, Back AL, et al. Interprofessional communication skills training for serious illness: evaluation of a small-group, simulated patient intervention. *J Palliat Med.* 2014;17(2):159-166.

| **Entry** | **Judgement** | **Support for judgement** |
| --- | --- | --- |
| Random sequence generation (selection bias) | High risk | Quote: “We report a before-after study…”  Comment: No randomization - before/after self control. |
| Allocation concealment (selection bias) | High risk | Quote: “We report a before-after study…”  Comment: No randomization - before/after self control. |
| Blinding of participants and personnel (performance bias) | High risk | Quote: “We report a before-after study of an interprofessional, simulation based workshop (Codetalk).” (and) “As part of this study, trainees… completed pre-intervention and post-intervention evaluative encounters with standardized patients.”  Comment: Impossible to blind participants from participation in workshop or SP encounters. |
| Blinding of outcome assessment (detection bias) | Low risk | Quote: “All SP sessions were digitally recorded and analyzed by trained coders.” (and) “The coders were blinded to whether the encounter occurred pre- or post-intervention.”  Comment: Analysis performed by third party observers. |
| Incomplete outcome data addressed (attrition bias) | High risk | Quote: “184 were randomized to the intervention. Of these, 145 (79%) completed audiorecorded interviews both pre- and post-intervention.”  Comment: Greater than 20% attrition rate. |
| Selective reporting (reporting bias) | Unclear risk | Comment: Study protocol not available. |
| Other Bias:  (maturation bias) | Unclear risk | Quote: “This evaluation is a before-and-after study, therefore cannot determine that the intervention caused improvement, but only that trainees improved over the time period during which the intervention occurred. However, since we found no association with year of training, a learning effect from one month of usual training seems less likely and is consistent with other studies indicating that clinician communication skills do not improve with time alone.”  Comment: Likely limited in that participants were in several different stages of training and course only occurred over a one month time frame. |
| Other Bias:  (Hawthorne effect) | High risk | Quote: “As part of this study, trainees… completed pre-intervention and post-intervention evaluative encounters with standardized patients.”  Comment: Participants were aware of participation in study and evaluation by a SP. Likely a tendency for people to perform better when being observed and when they are aware that they are in a study. |

**Journal Article:**

Tang L, Pang Y, Wang Y, He Y, Song L. Preliminary effects of oncology balint workshop in China. *Psycho-Oncology* 2014;23 SUPPL. 3 303-304.

| **Entry** | **Judgement** | **Support for judgement** |
| --- | --- | --- |
| Random sequence generation (selection bias) | High risk | Comment: Before/after self control - no randomization. It is also unclear who the participants were (other than Chinese oncologists) or how they were chosen. |
| Allocation concealment (selection bias) | High risk | Comment: Before/after self control - no randomization. |
| Blinding of participants and personnel (performance bias) | High risk | Comment: Impossible to blind participation in Balint group or completion of JSPE. |
| Blinding of outcome assessment (detection bias) | High risk | Comment: All participants would be aware of completion of the JSPE, a self-reported outcome measure. |
| Incomplete outcome data addressed (attrition bias) | Unclear risk | Quote: “Totally 28 participants took part in.”  Comment: It is not clear if all 28 participants filled out the pre and post JSPE. |
| Selective reporting (reporting bias) | Unclear risk | Comment: Study protocol not available. |
| Other Bias:  (social desirability bias) | Unclear risk | Quote: “The scores of pre-assessment on empathy and burnout were quite good.”  Comment: While there was no difference in pre and post JSPE, participants may have tended towards responding in a way that would be viewed more favorably in the pre-test leading to a smaller than expected change in post-test. |
| Other Bias:  (maturation bias) | Unclear risk | Quote: “The participants were assessed by JSPE… before (pre-assessment) and 6 months after the workshop (post-assessment.”  Comment: There is potential for improvement over 6 months due to maturation, though if this were the case, we would expect to see a difference in JSPE. Also, is is unclear how much experience the participating physicians have - results would also be minimized with more experience physicians. |
| Other Bias:  (Hawthorne effect) | Unclear risk | Comment: While participants would be aware of participant in the Balint workshop, it is unclear if they were aware that they were part of a study. If so, this could potentially alter behavior. However, if this were the case, we would expect to see a difference in JSPE. |
| Other Bias:  (small sample size) | Unclear risk | Comment: Small sample size could mean not having enough power to detect a statistically significant difference in the JSPE. |

**Journal Article:**

Yang KT, Yang JH. A study of the effect of a visual arts-based program on the scores of Jefferson Scale for Physician Empathy. *BMC Med Educ.* 2013;13:142.

| **Entry** | **Judgement** | **Support for judgement** |
| --- | --- | --- |
| Random sequence generation (selection bias) | High risk | Quote: “Recruitment of participants in this study was through convenience sampling. From 2010 October to 2011 January, all 98 medical students in clerkship and 22 PGY1s at our hospital participated in a required visual arts-based program. Among them 113...were recruited into this study.”  Comment: No randomization - before/after self control. |
| Allocation concealment (selection bias) | High risk | Quote: “From 2010 October to 2011 January, all 98 medical students in clerkship and 22 PGY1s at our hospital participated in a required visual arts-based program. Among them 113...were recruited into this study.”  Comment: No randomization - before/after self control. It is unclear why only 113 were recruited of the 120 students required to participate in the study and whether or not this would have been predictable to participants. |
| Blinding of participants and personnel (performance bias) | High risk | Quote: “In this study, the 4-hr program was divided into two 2-hr sessions (each one week apart). Both recruited and not recruited might attend the same group.” (and) “Students recruited were asked to complete both [pre & post JSPE & program assessment] while not recruited to complete at least the program assessment. Those not recruited were informed that they may complete the JSPE but their scores would not be included in the analysis.”  Comment: Impossible to blind participants from participation in program. Again, unclear why only 113 were recruited of the 120 students required to participate in the study but it seems that the recruited versus non-recruited would know which group they were in based on what they were asked to fill out. |
| Blinding of outcome assessment (detection bias) | High risk | Comment: Participants would all be aware of method of assessment (JSPE), a self reported outcome measure. |
| Incomplete outcome data addressed (attrition bias) | Low risk | Quote: “113 (95 clerks and 18 PGY1s) were recruited into this study.” (and) “There were 3 of the recruited unable to complete the 2-h sessions within 1 week as planned. The data were excluded from analysis.”  Comment: Low attrition rate. |
| Selective reporting (reporting bias) | Unclear risk | Comment: Study protocol not available. |
| Other Bias:  (social desirability bias) | Unclear risk | Comment: Potential for learners to want to answer questions in a way that would be viewed favorably by others, though with no statistically significant difference in pre- and posttest JSPEs, this is unlikely to have had much of an effect. |
| Other Bias:  (Hawthorne effect) | Unclear risk | Comment: Knowledge of participation in a study has the potential to improve performance/reporting, but if this were the case, we would have expected to see a difference in pre-/post-JSPE. |

**Journal Article:**

Gibon AS, Merckaert I, Lienard A, et al. Is it possible to improve radiotherapy team members’ communication skills? A randomized study assessing the efficacy of a 38-h communication skills training program. *Radiother Oncol.*  2013;109(1): 170-7.

| **Entry** | **Judgement** | **Support for judgement** |
| --- | --- | --- |
| Random sequence generation (selection bias) | Low risk | Quote: “The efficacy of the communication skills training program was assessed in a study allocating teams randomly… according to a computer generated randomization list.”  Comment: Adequate generation of randomized sequence. |
| Allocation concealment (selection bias) | Low risk | Quote: “The efficacy of the communication skills training program was assessed in a study allocating teams randomly… according to a computer generated randomization list.”  Comment: No way to foresee allocation with a computer generated randomization. |
| Blinding of participants and personnel (performance bias) | High risk | Quote: “51 were in the training group.”  Comment: Impossible to blind participants from participation in training program. |
| Blinding of outcome assessment (detection bias) | Low risk | Quote: “The audiotapes of the encounters were transcribed. Transcripts were analyzed by the LaComm, a French communication content analysis software.”  Comment: Utterances types (including empathy) were analyzed by a computer. |
| Incomplete outcome data addressed (attrition bias) | High risk | Quote: “A total of 96 team members registered for the training program… (n=65)... in the training group and … (n=31)... in the waiting list group. Sixteen… were excluded from the analysis for the following reasons: leaving the team between the assessment and training periods (n=1), drop-out (n=3), lack of patient contact (n=9), recording problem (n=2) and lack of training attendance.” (and) “Eighty team members completed the two encounters… of these team members, 51 were in the training group, and 29 were in the waiting list group.”  Comment: Decent size attrition rate for a smaller study and larger attrition rate in training group than waiting list group. |
| Selective reporting (reporting bias) | Unclear risk | Comment: Study protocol not available. |
| Other Bias:  (Hawthorne effect) | High risk | Comments: Awareness of participation in the study has the potential to improve performance. |
| Other Bias:  (chance bias) | Unclear risk | Quote: “More of the untrained team members were single (p=0.002)”  Comment: Potential for difference between trained and untrained team members due to baseline difference in marital status. |

**Journal Article:**

Johnson LA, Gorman C, Morse R, Firth M, Rushbrooke S. Does communication skills training make a difference to patients' experiences of consultations in oncology and palliative care services? *Eur J Cancer Care (Engl).* 2013;22(2):202-209.

| **Entry** | **Judgement** | **Support for judgement** |
| --- | --- | --- |
| Random sequence generation (selection bias) | High risk | Comment: There was no randomization. |
| Allocation concealment (selection bias) | High risk | Quote: “Senior HCPs working within oncology and palliative care services across the NECN who were required to attend the ACST course were invited to participate in this research.Those who were due to attend the ACST course with the time-period of the research were recruited for the ‘ACST group’. The ‘control group’ was recruited from the waiting list provided by the NECN.” (and) “Patients were excluded from being asked to complete the questionnaires if the HCP felt they were emotionally distressed.”  Comment: Group allocation unconcealed and by convenience. Study participants (HCP) could select which patients received questionnaires and some HCPs enrolled twice as many patients as other HCP, which likely influenced results. In addition, it is not clear if some patients participated more than once, which may biased results. |
| Blinding of participants and personnel (performance bias) | High risk | Quote: “Healthcare professionals who agreed to take part in the study were instructed to collect questionnaire data following each patient consultation. Patients aged 16 years and over were invited to take part in the study by their HCP following their consultation. Patients who agreed were either given a research pack by the HCP explaining the nature and purpose of the project.”  Comment: From the authors description it seems that the HCP and the patients grading their performance were aware of the purpose of the study to measure. |
| Blinding of outcome assessment (detection bias)  CARE measure | Unclear risk | Quote: “All questionnaire responses were anonymous and patients were assured that their HCP would not be able to identify them.”  Comment: The patients evaluating their physicians’ empathy were not aware of physician course attendance (blinded), but the protocol is not clear on who collected patient questionnaires and whether or not that individual was blinded as to group allocation of the physician. |
| Incomplete outcome data addressed (attrition bias) | High risk | Quote: “A total number of 46 HCPs agreed to take part in the study. Twelve dropped out of the study (nine from the ACST group and three from the control group). This dropout was due to time pressures in clinics or the HCP felt they would be unable to see sufficient patients in the time period. There were 13 HCPs who did not obtain a minimum of 20 completed CARE measures and so were not included in the final analysis.”  Comment: The authors report that it is recommended that a minimum of 40 patients are required to complete a CARE for reliable results. They sought to get 25 questionnaires per HCP and in end included HCPs with 20 surveys completed with a high study attrition rate. There were a variable number of surveys in each group at each time point. |
| Selective reporting (reporting bias) | Unclear risk | Quote: “It was hypothesised that scores on the patient-rated CARE measure will reflect greater clinician relational empathy following completion of the 3-day ACST course, when compared with a control group who have yet to complete the ACST course.”  Comment: Authors describe their protocol and outcome measures. In their analysis they explore and report additional non-prespecified outcomes. |
| Other Bias:  (Hawthorne effect) | High risk | Comment: Participants may have known or been able to discern that their empathy improvement was being tested. Tendency to perform better when participating in an experiment may have lead to higher test scores. |
| Other Bias:  (small sample size) | Unclear risk | Quote: “21 HCPs of which 12 were in the ACST group and 9 were the control group.”  Comment: Very few participating HCP and they collected varying numbers of questionnaires. |

**Journal Article:**

Blanco MA, Maderer A, Price LL, Epstein SK, Summergrad P. Efficiency is not enough; you have to prove that you care: role modelling of compassionate care in an innovative resident-as-teacher initiative. *Educ Health (Abingdon).* 2013;26(1):60-65.

| **Entry** | **Judgement** | **Support for judgement** |
| --- | --- | --- |
| Random sequence generation (selection bias) | High risk | Quote: “Programme Directors forwarded the invitation to participate in the programme to their residents; total population of approximately 400 residents. Residents were promised that by participating in the programme they would: be trained in practicing and teaching compassionate, relationship-centred care through role modelling; develop and deliver a scholarly communication about their experience with the programme to their peers at their sites and add this scholarly work to their curriculum vitae; and receive a US$500 honorarium. The final pool of participants consisted of 41 residents across the six sites, representing seven specialties:”  Comment: There was no randomization. |
| Allocation concealment (selection bias) | High risk | Quote: “The final pool of participants consisted of 41 residents across the six sites, representing seven specialties:”  Comment: All participants were volunteers for the course. Allocation was unconcealed. There was no randomization, and no control arm. |
| Blinding of participants and personnel (performance bias) | Unclear risk | Quote: “Residents were promised that by participating in the programme they would: be trained in practicing and teaching compassionate, relationship-centred care through role modelling; develop and deliver a scholarly communication about their experience with the programme to their peers at their sites and add this scholarly work to their curriculum vitae; and receive a US$500 honorarium.”  Comment: There authors do not present whether or not the participants were blinded. |
| Blinding of outcome assessment (detection bias)  Interpersonal and Communication Skills performance on standardized patient encounter.  JSPE | High risk | Comment: The authors do not describe any blinding of outcome assessments. |
| Incomplete outcome data addressed (attrition bias) | High risk | Quote: “For quantitative analytical purposes, we used a subset of the sample of participating residents based on the number of participants who completed all the questions so that we could calculate total scores.”  Comment: Portions of the study were not completed by all participants. |
| Selective reporting (reporting bias) | Unclear risk | Quote: “In this brief communication, we describe the programme implementation and main short-term outcomes of our evaluation of the programme.” (and) “We anticipated that resident’s performances on the SP exercises and questionnaires would improve after their participation in the programme. We also expected that journal writing would raise resident’s awareness of their demonstrations or failures to demonstrate compassionate, relationship-centred care in their daily practice.”  Comment: The authors do not specify their main outcome measures and leave subjectivity in their plan for evaluating outcome. |
| Other Bias:  (other) | Unclear risk | Comment: The students who elected to participate in the training may have been more motivated to learn and master the empathy / compassionate care material. |
| Other Bias:  (other) | Unclear risk | Comment: Results of pre- and post testing are attributed to the course, but there was no control arm of students matriculating through the semester without this course. |

**Journal Article:**

Riess H, Kelley JM, Bailey RW, Dunn EJ, Phillips M. Empathy training for resident physicians: a randomized controlled trial of a neuroscience-informed curriculum. *J Gen Intern Med.* 2012;27(10):1280-1286.

| **Entry** | **Judgement** | **Support for judgement** |
| --- | --- | --- |
| Random sequence generation (selection bias) | Low risk | Quote: “Participating physicians were randomly assigned in a 1:1 allocation ratio to either the training intervention or to standard residency or fellowship training. Group assignment was determined by a computer-generated random number sequence.”  Comment: Adequate randomization. |
| Allocation concealment (selection bias) | Unclear risk | Comment: Concealment is not described. |
| Blinding of participants and personnel (performance bias) | Unclear risk | Comment: By study protocol participants would necessarily be aware that they were enrolled in an empathy training course. |
| Blinding of outcome assessment (detection bias) CARE measure | Low risk | Quote: “Patients were blind to physician randomization, and physicians were blinded to which patients completed the surveys.”  Comment: Patients blinded as to whether or not physician was in empathy training when filling out the CARE measure. |
| Blinding of outcome assessment (detection bias) JSPE | High risk | Comment: It is impossible to blind participants in filling out the JSPE, a self-reported outcome measure. |
| Incomplete outcome data addressed (attrition bias) | Low risk | Quote: “Each physician was rated by multiple patients (pre mean 4.6±3.1; post mean 4.9±2.5) to minimize the impact of idiosyncratic ratings and increase reliability”  Comment: The authors report data for all participants for the primary outcome. |
| Selective reporting (reporting bias) | Low risk | Quote: “The primary outcome measure was change in empathic and relational skills as assessed by patients blinded to physician randomization. Patients rated physicians on the Consultation and Relational Empathy Measure (CARE).”  Comment: Protocol is available and primary and secondary outcomes are pre-specified. |
| Other Bias:  (Hawthorne effect) | Unclear risk | Participants may have known or been able to discern that their empathy improvement was being tested. Tendency to perform better when participating in an experiment may have lead to higher test scores. |
| Other Bias:  (other) | Unclear risk | Comment: The students who elected to participate in the training may have been more motivated to learn and master the empathy / compassionate care material |
| Other Bias:  (other) | Unclear risk | Comment: The authors do not report median number of CARE measures that were done for each physician, one physician with multiple vs others with only one might have skewed results. |

**Journal Article:**

Cinar O, Ak M, Sutcigil L, et al. Communication skills training for emergency medicine residents. *Eur J Emerg Med.* 2012;19(1):9-13.

| **Entry** | **Judgement** | **Support for judgement** |
| --- | --- | --- |
| Random sequence generation (selection bias) | High risk | Quote: “The study included all emergency medicine residents in the GATA Emergency Medicine Department (n = 20).”  Comment: There was no randomization. |
| Allocation concealment (selection bias) | High risk | Comment: There was no randomization, and no control arm; all residents participated in this empathy training |
| Blinding of participants and personnel (performance bias) | High risk | Quote: “After obtaining approval from the local ethics committee, a psychoeducation program that was intended to improve communication and empathy skills in emergency medicine residents was held for 6 weeks (90 min per week).”  Comment: The authors do not report any blinding of participants to the purpose of the study |
| Blinding of outcome assessment (detection bias)  Increase in respect, kindness, and understanding as assessed by patients | Unclear risk | Quote: “Patient satisfaction surveys on the physician–patient relationship were filled out by patients who presented to the ED within the previous 3 months (September 2009–October 2009) and after completion of the program (January 2010–April 2010).”  Comment: The authors do not report any blinding, but the patients filling out surveys likely did not know about the empathy training. |
| Blinding of outcome assessment (detection bias)  Empathy Quotient | High risk | Comment: This is a self report survey and therefore participants could not be blinded. |
| Incomplete outcome data addressed (attrition bias) | Unclear risk | Comment: The authors do not report whether or not all participants completed all portions of the study; ie., whether or not there were patient surveys for each participant. |
| Selective reporting (reporting bias) | Unclear risk | Quote: “The comparison of pretraining and post-training scores on communication skills, empathy, and patient satisfaction is presented in Table 1.”  Comment: Protocol not available. Outcome measures reported not all pre-specified. |
| Other Bias  (Small sample size) | High risk | Quote: “Twenty emergency medicine residents from the GATA Emergency Medicine Department participated”  Comment: Small sample size limits generalizability. |
| Other Bias  (Hawthorne) | Unclear risk | Comment: Participants may have known or been able to discern that their empathy improvement was being tested. Tendency to perform better when participating in an experiment may have lead to higher test scores. |

**Journal Article:**

Ozcan CT, Oflaz F, Bakir B. The effect of a structured empathy course on the students of a medical and a nursing school. *Int Nurs Rev.* 2012;59(4):532-538.

| **Entry** | **Judgement** | **Support for judgement** |
| --- | --- | --- |
| Random sequence generation (selection bias) | High risk | Quote: “This study had a pre-/post-test quasi-experimental design.” (and) “First year students of nursing and medical schools of the same university were included in the study.”  Comment: There was no randomization. |
| Allocation concealment (selection bias) | High risk | Quote: “First year students of nursing and medical schools of the same university were included in the study.” (and) “Of the 257 students in total, 143(63.3%) medical and 83 (36.7%) nursing students completed the questionnaire successfully before the empathy course (226, 88%).”  Comment: All first year students were invited to complete a questionnaire. Allocation was on volunteer basis. |
| Blinding of participants and personnel (performance bias) | High risk | Quote: “The purposes and methods of study were explained to the students. After receiving their verbal consent, the study was conducted with volunteer students.”  Comment: Students electing to participate may have been more interested and motivated to improve their ability to employ empathy. |
| Blinding of outcome assessment (detection bias)  Increase in Empathetic Communication Skill Scale and the Empathetic Tendency Scale | Low risk | Quote: “The students were asked to choose their own nickname to compare their pre-test scores with their post-test scores. Non-teaching personnel administered the scales and questionnaires to the students.”  Comment: Scorers blinded. |
| Incomplete outcome data addressed (attrition bias) | Unclear risk | Comment: The authors did not report how many of the participants completed the study. They directed the reader to Figure 3 for results. It was not clear from Figure 3 in the on-line supplement how many participants withdrew from the study. |
| Selective reporting (reporting bias) | Unclear risk | Quote: “(objective)**1** The appropriate empathy course has a positive effect on empathic skills and tendency of all first year students of a medical and a nursing school. **2** Empathic skills and tendency differ according to female and male groups.”  Comment: The study protocol is explained and outcomes are reported but not in a pre-specified manner. |
| Other Bias:  (maturation bias) | Unclear risk | Comment: Natural change/improvement in time may have lead to higher post test scores. |
| Other Bias:  (Hawthorne effect) | Unclear risk | Comment: The tendency to perform better when participating in an experiment may have lead to higher post test scores. |
| Other Bias:  (Gender bias, selection bias) | Unclear risk | Quote: “There are no male students in the nursing school.” (and) “In the study, all included medical students were male (100%).”  Comment: The authors are seeking to determine effects on female and male groups while all participating medical students were men and all nursing students were women. The difference in their elected field of study may have biased results and findings may not have been related to gender. |

**Journal Article:**

Lim BT, Moriarty H, Huthwaite M. "Being-in-role": A teaching innovation to enhance empathic communication skills in medical students. *Med Teach.* 2011;33(12):e663-669.

| **Entry** | **Judgement** | **Support for judgement** |
| --- | --- | --- |
| Random sequence generation (selection bias) | High risk | Quote: “Year 2009 students served as the control group while the Year 2010 students were the intervention group in this study.”  Comment: There was no randomization. |
| Allocation concealment (selection bias) | High risk | Quote: “Year 2009 students served as the control group while the Year 2010 students were the intervention group in this study.”  Comment: The allocation was unconcealed. |
| Blinding of participants and personnel (performance bias) | High risk | Quote: “They also received a briefing about the education research project and participant informed consent was sought from them”  Comment: Participants were not blinded. |
| Blinding of outcome assessment (detection bias)  JSPE | High risk | Quote: “A key ethical consideration was blinding of the tutors and student assessors to the students who were or were not participants”  Comment: JSPE is a self reported outcome measure |
| Incomplete outcome data addressed (attrition bias) | Unclear risk | Quote: “Analysis of the difference in pre- and post-intervention empathy scores for the control and intervention groups”  Comment: The authors do not comment on whether all participants completed each portion of the study. |
| Selective reporting (reporting bias) | Unclear risk | Quote: “the empathy level of medical students in the intervention group (as measured by JSPE) would increase following the teaching innovation (drama training).”  Comment: Protocol is not available. Primary outcome measure is pre-specified. |
| Other Bias  (Hawthorne effect) | Unclear risk | Comment: The tendency to perform better when participating in an experiment may have lead to higher post test score |

**Journal Article:**

Tulsky JA, Arnold RM, Alexander SC, et al. Enhancing communication between oncologists and patients with a computer-based training program: a randomized trial. *Ann Intern Med.* 2011;155(9):593-601

| **Entry** | **Judgement** | **Support for judgement** |
| --- | --- | --- |
| Random sequence generation (selection bias) | Unclear risk | Quote: “The SCOPE (Studying Communication in Oncologist– Patient Encounters) Trial was a single-blind, randomized, controlled, parallel-group study.”  Comment: The authors do not describe the method used to generate the group allocation. |
| Allocation concealment (selection bias) | Unclear risk | Quote: “We sent patients recruitment letters that were signed by their oncologists, and we met them before their recorded visit to obtain consent and conduct a baseline survey.”  Comment: Method of concealment not described. |
| Blinding of participants and personnel (performance bias) | High risk | Quote: “Trial was a single-blind, randomized, controlled, parallel-group study.”  Comment: The coders were blinded. It is not clear how the study recruitment described the study to the physicians and patients, but by study design the oncologist physicians were not blinded as they knew if they were receiving extra training in empathy. |
| Blinding of outcome assessment (detection bias)  Number empathetic statements  Patient perceived trust  Perceived patient | Low risk | Quote: “Two independent, blinded coders were trained over 6 weeks.”  Comment: coders blinded |
| Incomplete outcome data addressed (attrition bias) | Unclear risk | Comment: Study flow diagram describes the included subjects. Not all subjects completed all portions of the study (missing all surveys and post visits). |
| Selective reporting (reporting bias) | Low risk | Quote: ”We report the results of a randomized, controlled trial that tested the effectiveness of this intervention in improving oncologist behavior. A secondary objective was to evaluate the effect of the intervention on patients’ perceptions of their oncologists. Our primary outcome measures from the recordings were empathic statements and responses to empathic opportunities, both of which we referred to as emotion-handling skills.”  Comment: The study protocol is available and the authors reported outcomes in the pre-specified way. |
| Other Bias:  (social desirability bias) | Unclear risk | Quote: “patients completed the measures of trust, perceived empathy, therapeutic alliance, and perceived knowledge”  Comment: When asked to comment on their physicians, patients may have responded in a way that they perceived to be desirable to the investigator. |
| Other Bias:  (Hawthorne effect) | High risk | Comment: Participants were aware of participation in study and evaluation by a rater so there was likely a tendency for the physicians to perform better. |
| Other Bias:  (small sample size) | Unclear risk | Comment: Only 24 oncologists fully participated in each arm of the study which may limit generalizability. |

**Journal Article:**

Riess H, Kelley JM, Bailey R, Konowitz PM, Gray ST. Improving empathy and relational skills in otolaryngology residents: a pilot study. *Otolaryngol Head Neck Surg.* 2011;144(1):120-122.

| **Entry** | **Judgement** | **Support for judgement** |
| --- | --- | --- |
| Random sequence generation (selection bias) | High risk | Quote: “Eleven otolaryngology residents (43% female; mean age, 31) completed three 90-minute empathy and relational skills training modules, presented at 0, 4, and 6 weeks.”  Comment: There was no randomization. |
| Allocation concealment (selection bias) | High risk | Comment: There was no randomization, and no control arm; all residents participated in this empathy training. |
| Blinding of participants and personnel (performance bias) | High risk | Quote: “At baseline and at study completion, residents completed 5 self-report measures”  Comment: The participants were aware of what aspects of their behavior was to be measured and how. |
| Blinding of outcome assessment (detection bias)  BEES, JSPE, CARE measure | Unclear risk | Comment: There is no mention of blinding of the investigators making outcome assessments. JSPE is a self assessment. |
| Incomplete outcome data addressed (attrition bias) | Unclear risk | Comment: The authors did not indicate if all portions of the study were completed by each participant. |
| Selective reporting (reporting bias) | High risk | Quote: “To address this need, the first author developed a novel empathy training protocol based on the neurobiological and physiological mechanisms and interpersonal processes that improve empathy.” (and from Results) “Figure 1 and Supplemental Figure S1 (online) show statistically significant improvements in residents’ knowledge of the neurobiology and physiology of empathy.”  Comment: Study protocol not available. |
| Other Bias:  (social desirability) | High risk | Quote: “After the training was completed, residents reported agreement with the following statements: (1) I found this training to be interesting (83%), (2) I found this training to be helpful (100%), (3) I will be able to apply the concepts and skills I learned to clinical practice (100%), and (4) I am motivated to try some of the techniques I learned (83%).”  Comment: It is possible that in this small training program where the participant subjects were all residents that they answers were biased to please their supervisors. |
| Other Bias:  (small sample size) | High risk | Quote: “The authors tested the effectiveness of this protocol in a pilot study with 11 otolaryngology residents.”  Comment: Small sample size limits generalizability |

**Journal Article:**

Cahan MA, Larkin AC, Starr S, et al. A human factors curriculum for surgical clerkship students. *Arch Surg.* 2010;145(12):1151-1157.

| **Entry** | **Judgement** | **Support for judgement** |
| --- | --- | --- |
| Random sequence generation (selection bias) | High risk | Comment: There was no randomization of study participants |
| Allocation concealment (selection bias) | High risk | Quote: “The full-day human factors curriculum was presented to students in block 2 and block 4. Students in blocks 1 and 3 received the standard curriculum without human factors training.”  Comment: There was an unconcealed method of allocation. Participants were assigned to a study group based on which block they had the surgical rotation. |
| Blinding of participants and personnel (performance bias) | High risk | Quote: “Students in the intervention group were asked to complete a ‘Commitment to Change’ form and an evaluation of the program immediately after the training session. The control and intervention groups completed a series of human factors evaluation measures on the last day of the clerkship.”  Comment: Students in the intervention group may have been influenced by the commitment to change form which may bias them toward filling out the post rotation evaluation differently than they might have without this form; ie the difference found in the post test may be related to the commitment form rather than the course intervention. |
| Blinding of outcome assessment (detection bias)  5-point empathy score | Unclear risk | Quote: (Under patient communication vignette)  “Responses to the vignettes were randomly sorted and masked as to participation group, then coded.”  Comment: The authors report that coders were blinded as to whether or not subject had had the ‘patient communication’ training intervention, but blinding is not mentioned for coding of the ‘communication with team members’ and the ‘work-life balance’ vignettes. |
| Incomplete outcome data addressed (attrition bias) | Unclear risk | Quote: “4 brief video vignettes, each presenting a communication challenge included in the human factors curriculum.” (and) “Three hundred eighty-two responses to vignettes were analyzed, 190 from control students and 192 from intervention students.” (and in Pilot two) “Fifty students participated in the intervention in the second year of the pilot. Forty-four of the students, 24 from block 1 and 20 from block 2, completed all the evaluation measures at pretraining and post-course assessments. Only students with complete data at both time points are included in the results.”  Comment: There should have been 388 vignettes for analysis in Pilot one and the authors do not describe the missing data. Then for Pilot two the authors do not describe why some participants do not have pre and post course evaluations. It is unclear if this missing data would have affected study results. |
| Selective reporting (reporting bias) | Unclear risk | Quote: “Main Outcome Measure: Empathetic communication skill, teamwork, and patient safety attitudes and self reported use of time management strategies.” (and) “(responses were) coded using a previously developed 5-point scale”  Comment There is no reference to the validation of this scale. |
| Other Bias:  (Maturation bias) | Unclear risk | Quote: Students in block 1 and block 2 received the training and completed the same series of evaluation measures before training and at the end of the clerkship.  Comment: Natural change/improvement in time may have lead to higher post test scores. No control group to compare. |
| Other Bias:  (Hawthorne effect) | High risk | Comment: The tendency to perform better when participating in an experiment may have lead to higher post test scores. |

**Journal Article:**

Sripada BN, Henry DB, Jobe TH, Winer JA, Schoeny ME, Gibbons RD. A randomized controlled trial of a feedback method for improving empathic accuracy in psychotherapy. Psychol Psychother. 2011;84(2):113-127.

| **Entry** | **Judgement** | **Support for judgement** |
| --- | --- | --- |
| Random sequence generation (selection bias) | Low risk | Quote: “randomized controlled trial of a feedback intervention” (and) “Patient–therapist pairs were randomly assigned by the first author to the intervention or control group by flipping a coin.”  Comment: The investigators describe a random component in the sequence generation. |
| Allocation concealment (selection bias) | High risk | Quote: “Participating therapists screened potential patients for inclusion criteria.”  Comment: Investigators did not screen participants but the therapists who would be in the study as well selected the patients for participation. |
| Blinding of participants and personnel (performance bias) | Unclear risk | Quote: “Twelve psychiatry residents and 16 patients volunteered to participate in the study.”  Comment: There is no mention of blinding of participants to hypotheses and outcomes but the control group therapists was not able to be influenced by the questionnaires because they were blinded to the patient answers. |
| Blinding of outcome assessment (detection bias)  Empathetic accuracy  Barrett-Lennard empathy subscale | Unclear risk | Quote: “In the intervention condition, at the beginning of the next session, therapists and patients exchanged ratings from the preceding session, providing an opportunity to discuss their respective views.”  Comment: The protocol of the study called for therapists to see questions regarding patients’ perceptions of them and compare to their own impression of how the patients would answer the questions and then discuss. It is unclear if therapists were becoming more empathetic to patients or just able to estimate answers better based on viewing results week after week. |
| Incomplete outcome data addressed (attrition bias) | Unclear risk | Quote: “One potential participant declined participation and one patient withdrew from the study after three sessions, citing a change in insurance coverage as a reason. This person’s data were excluded from analyses.”  Comment: Not all participants’ data were included. |
| Selective reporting (reporting bias) | Low risk | Quote: “Table 2 reports the baseline and final scores on all outcome measures”  Comment: Study protocol is available and all outcome measures are reported. |
| Other Bias:  (small sample size) | Unclear risk | Quote: “This paper reports a pilot study involving a randomized controlled trial of a feedback intervention.”  Comment: The small sample size limits generalizability. |

**Journal Article:**

Ghetti C, Chang J, Gosman G. Burnout, psychological skills, and empathy: balint training in obstetrics and gynecology residents. *J Grad Med Educ.* 2009;1(2):231-235.

| **Entry** | **Judgement** | **Support for judgement** |
| --- | --- | --- |
| Random sequence generation (selection bias) | High risk | Comment: There was no randomization. |
| Allocation concealment (selection bias) | High risk | Quote: “Those residents who agreed to participate in the study completed the Maslach Burnout Inventory, the Psychological Medicine Inventory, and the Jefferson Scale of Physician Empathy prior to initiating the course and at 12 months.”  Comment: Study participants were a self selected group. It is not clear whether or not they understood the purpose of the study. Both of these factors may have biased results. |
| Blinding of participants and personnel (performance bias) | Unclear risk | Quote: “The investigators and faculty members leading the groups were not present at the time of recruitment and were blinded to which residents chose to participate.”  Comment: It is not clear whether or not participants knew the purpose of the study. |
| Blinding of outcome assessment (detection bias)  JSPE | High risk | Quote: “The investigators and faculty members leading the groups were not present at the time of recruitment and were blinded to which residents chose to participate.”  Comment: The investigators were blinded as to which residents participated at the time of recruitment, but JSPE is self-reported outcome measure so participants are aware if they had the training. |
| Incomplete outcome data addressed (attrition bias) | Unclear risk | Quote: “Complete data were available for 17 residents who completed both baseline and 12-month questionnaires.”  Comment: The authors do not report how many residents consented to the study and did not complete the protocol. |
| Selective reporting (reporting bias) | Low risk | Quote: “The aim of this study was to assess burnout, behavioral-medicine skills, and empathy among obstetrics and gynecology residents before and after implementation of this new curriculum by using validated measures.”  Comment: The authors describe their protocol and state that they will use validated measures to assess outcome measures. They specify these validated measures in the methods section. |
| Other Bias:  (small sample size) | High risk | Quote: “Seventeen residents completed baseline and 12- month questionnaires. (and) Participants included the majority of first- and third-year residents. Nonparticipants were primarily second- and fourth-year residents.”  Comment: Small sample size limits generalizability. |
| Other Bias:  (other) | Unclear risk | Comment: The timing of the study and Balint training during residency of the potential subjects (1st, 2nd, 3rd or 4th year) may have influenced their willingness to participate which could also correlate to burnout and emotional exhaustion. |

**Journal Article:**

Bonvicini KA, Perlin MJ, Bylund CL, Carroll G, Rouse RA, Goldstein MG. Impact of communication training on physician expression of empathy in patient encounters. *Patient Educ Couns.* 2009;75(1):3-10.

| **Entry** | **Judgement** | **Support for judgement** |
| --- | --- | --- |
| Random sequence generation (selection bias) | Low risk | Quote: “Physicians who volunteered for the study were randomized into one of four experimental treatment groups in a fully crossed 2 × 2 between-subjects analysis of variance (ANOVA) ...No physicians who volunteered were excluded, and successive groups of 24 physicians were randomized to one of four conditions (physician trained, patient trained, physician and patient trained, neither physician nor patient trained) using a computer-generated random order. Physician training was delivered in small groups and office data collection depended upon the scheduling of research assistants. “  Comment: Quote from the original study protocol, Haskard KB, et al. Physician and patient communication training in primary care: effects on participation and satisfaction health psychology. Health Psychology, 27(5) Seo2008, 513-522. Physic |
| Allocation concealment (selection bias) | Low risk | Quote: “A random sample (n = 232) of audiotaped physician–patient interactions was drawn from the original dataset of the randomized control trial for recoding using a second measurement, Empathy Communication Coding System (ECCS) [33,34]. Audiotapes chosen for analyses were randomly selected from among the audiotapes from the original dataset for each study physician (n = 160) at each of the two assessment time points.”  Comment: Random sampling. |
| Blinding of participants and personnel (performance bias) | High risk | Quote: “Physician empathic expression (the dependent variable) was measured at two time points, during patient encounters before communication training (baseline) and during patient encounters 6 months after training was completed.”  Comment: participants were aware of whether or not they had the empathy training |
| Blinding of outcome assessment (detection bias)  Third party observer using ECCS and GRS | Low risk | Quote: “160 doctors and their patients were coded using the Global Rating Scale (GRS), a third-party coding instrument used for measuring overall ratings of physician empathy.”  Comment: Coding was performed by third party without knowledge of empathy training |
| Incomplete outcome data addressed (attrition bias) | Unclear risk | Quote: “Each of the 232 coded interactions contained three ratings and a single score for the variable of global empathy (GRS), calculated by summing the three ratings.”  Comment: All data accounted for in this portion of the study, but it is an analysis of video-taped encounters from an original study that randomized 156 physicians and 10 were lost to follow-up. |
| Selective reporting (reporting bias) | Low risk | Comment: Study protocol is available and the study’s pre-specified outcomes are reported |
| Other Bias  (Hawthorne effect) | High risk | Comment: The tendency to perform better when participating in an experiment may have lead to higher post test scores |

**Journal Article:**

Shapiro SM, Lancee WJ, Richards-Bentley CM. Evaluation of a communication skills program for first-year medical students at the University of Toronto. *BMC Med Educ.* 2009;9:11.

| **Entry** | **Judgement** | **Support for judgement** |
| --- | --- | --- |
| Random sequence generation (selection bias) | Unclear risk | Quote: “The study used a repeated measures design with a waiting list control group: students were randomly assigned to groups starting the educational intervention in either September (N = 38) or February (N = 41), with the latter being used as a control for the former.”  Comment: Unclear how they were randomized. |
| Allocation concealment (selection bias) | Unclear risk | Quote: “After providing informed written consent and completing the screening procedure, students were randomly assigned to groups starting the educational intervention in either September or February.”  Comment: The authors do not describe the process of a concealed allocation. |
| Blinding of participants and personnel (performance bias) | High risk | Quote “:...randomly assigned to groups starting the educational intervention in either September or February.”.  Comment: Subjects knew which group they were in based on when they had the course. |
| Blinding of outcome assessment (detection bias)  Self Assessment of Interpersonal Competence Questionnaire  Standardized patient assessment using Interpersonal Skills Rating Scale | Unclear risk | Comment: A portion was self-assessment which could not be blinded and it is unclear if standardized patients were blinded as to which group they were assessing. . |
| Blinding of outcome assessment (detection bias)  Staff-Patient Interaction rating scale | Low risk | Quote:“Responses were scored by raters who were blind to the randomization, the identity of the participant and concurrent other responses by a participant.”  Comment: Outcome assessors blinded |
| Incomplete outcome data addressed (attrition bias) | Unclear risk | Quote: “12 participants who lost interest while on the waiting list were not included in the above analysis as intend-to-treat participants because their inclusion as no-change controls would inflate the contrast between the two groups.”  Comment: Did not include all subjects in final analysis. |
| Selective reporting (reporting bias) | Unclear risk | Quote: “The purpose of this study was to evaluate the effectiveness of the University of Toronto's Therapeutic Communication Program (TCom) at improving first-year medical students' communication skills” and “It was hypothesized that, compared to waiting list control participants, group supervision participants would have a greater improvement in their therapeutic communication skills over the time of the intervention.”  Comment: The study protocol is not available. The authors did not pre-specify primary outcome measure. |
| Other Bias  (other) | High risk | Quote:” informed student self-selection beforehand: the program is offered to students on a voluntary basis and they are provided with a careful description of the program's expectations and challenges.”  Comment: Students motivated to complete additional communication training elected to enroll in this course. Limits generalizability. |

**Journal Article:**

Fernndez-Olano C, Montoya-Fernndez J, Salinas-Snchez AS. Impact of clinical interview training on the empathy level of medical students and medical residents. *Med Teach.* 2008;30(3):322-324.

| **Entry** | **Judgement** | **Support for judgement** |
| --- | --- | --- |
| Random sequence generation (selection bias) | High risk | Quote: “We present a quasi-experimental study of 203 subjects.”  Comment: There was no randomization. Participant group was assigned by hospital location. Allocation appears to have been by location of intervention. |
| Allocation concealment (selection bias) | High risk | Quote: “All participants answered the JSPE questionnaire without being told that the study was ending, with pre- and post workshop evaluations.”  Comment: While participants may not have known that they would have a post workshop evaluation, the investigators could for see participant group allocation. The group allocation appears to have been by location of intervention. |
| Blinding of participants and personnel (performance bias) | Unclear risk | Quote: “All participants answered the JSPE questionnaire without being told that the study was ending, with pre- and post workshop evaluations not less than 7 days apart and not more than 20 days.”  Comment: It is unclear if participants understood the purpose of the study during the intervention (the workshop) and the post- workshop evaluation. |
| Blinding of outcome assessment (detection bias)  JSPE | High risk | Quote: “This study assesses the impact of a communication skill workshop on medical students’ and medical residents’ empathy level, using the Jefferson Scale of Physician Empathy (JSPE) (Hojat et al. 2002a) to measure empathy level.”  Comment: The scale on this is a numeric scale, so it is unclear that outcome assessors could bias study results just by being unblinded to study group allocation. JSPE is by self-report though, so outcome assessment is necessarily unblinded. |
| Incomplete outcome data addressed (attrition bias) | Unclear risk | Quote: “The subjects were divided into a control group with 75 individuals (20 residents/55 students) and an experimental group composed of 128 individuals (46 residents/82 students).”  Comment: The authors do not report study attrition or address whether or not there are missing data. |
| Selective reporting (reporting bias) | Unclear risk | Quote: “This study assesses the impact of a communication skill workshop on medical students’ and medical residents’ empathy level, using the Jefferson Scale of Physician Empathy (JSPE) (Hojat et al. 2002a) to measure empathy level.”  Comment: There is no pre-specified primary outcome. |
| Other Bias | Unclear risk | Comment: Insufficient information to assess whether an important risk of bias exists |

**Journal Article:**

Dow AW, Leong D, Anderson A, Wenzel RP, Team VCUT-M. Using theater to teach clinical empathy: a pilot study. *J Gen Intern Med.* 2007;22(8):1114-1118.

| **Entry** | **Judgement** | **Support for judgement** |
| --- | --- | --- |
| Random sequence generation (selection bias) | High risk | Quote: “The study was neither randomized nor blinded.”  Comment: |
| Allocation concealment (selection bias) | High risk | Quote: “The study was neither randomized nor blinded.” (and) “All of the residents who were participating in a month-long Ambulatory Care Teaching Block were placed in the intervention group (n=14). Six other residents, selected because they had ambulatory continuity clinic at the same time and location, comprised a control group. Participants were instructed about the study processes and its goals and given the option to participate.”  Comment: This study employed an explicitly unconcealed procedure for group selection. |
| Blinding of participants and personnel (performance bias) | High risk | Quote: “The study was neither randomized nor blinded.”  Comment: Behavior may have been influenced solely by participants knowledge of the purpose of the study. |
| Blinding of outcome assessment (detection bias)  Increased empathetic communication  Verbal communication | High risk | Quote: “The study was neither randomized nor blinded.”  Comment: Evaluators of empathy were not blinded to participants’ group allocation |
| Incomplete outcome data addressed (attrition bias) | Low risk | Comment: It appears that all participants completed the study and that the authors reported the data from their participation. |
| Selective reporting (reporting bias) | Unclear risk | Quote: “The purpose of this present study, therefore, was to determine whether professors from the department of theater could teach and assess clinical empathy to Internal Medicine residents.”  Comment: Authors clearly stated objective but not how they define success of whether professors from the department of theater could teach and assess clinical empathy to Internal Medicine residents. |
| Other Bias:  (Hawthorne effect) | Unclear risk | Comment: The tendency to perform better when participating in an experiment may have lead to higher post test scores. |
| Other Bias:  (small sample size) | High risk | Quote: “Twenty Internal Medicine residents: 14 in the intervention group, 6 in the control group.” (and) “One of the 4 members of the Department of Theater performed the evaluation via a modified scoring tool commonly used within the Theater Department to assess student performance.”  Comment: The small sample size limits generalizability. Also, it is not clear what the agreement the 4 members of the Department of Theater have in assessing participants. |

**Journal Article:**

Cataldo KP, Peeden K, Geesey ME, Dickerson L. Association between Balint training and physician empathy and work satisfaction. *Fam Med.* 2005;37(5):328-331.

| **Entry** | **Judgement** | **Support for judgement** |
| --- | --- | --- |
| Random sequence generation (selection bias) | High risk | Quote: “Residents were given the option to participate in Balint training or not participate. Group one consisted of 113 physicians who had regularly participated in Balint training. (And) Group two consisted of 69 physicians who chose not to attend Balint sessions after a 6-month mandatory period.”  Comment: Participants were self selected to the Balint training. No randomization. |
| Allocation concealment (selection bias) | High risk | Comment: It is not clear whether or not participants knew the purpose of the study. But, the authors knew to which group participants were assigned from the onset. Allocation was unconcealed. |
| Blinding of participants and personnel (performance bias)  JSPE | High risk | Quote: “Participants were family physicians who graduated from the Medical University of South Carolina (MUSC) Family Medicine Residency Program between the years of 1982 and 1999. The questionnaire consisted of two separate surveys. The first was the Jefferson Scale of Physician Empathy (HP-Version R), a validated quantitative measurement used to assess levels of physician empathy.”  Comment: It is not clear whether or not participants knew the purpose of the study but JSPE is a self report measure so would be unblinded. |
| Blinding of outcome assessment (detection bias) | Unclear risk | Quote: “The first was the Jefferson Scale of Physician Empathy (HP-Version R), a validated quantitative measurement used to assess levels of physician empathy.11 It contains 20 statements, with a 7-point scale that measures the extent of agreement to the statement. The second instrument was a validated physician Work Satisfaction Survey, developed by Hueston, with a 5-point scale.”  Comment: There was no blinding of outcome assessment but it is not clear that this would influence analysis of surveys using numeric scales that did not allow for any subjective interpretation of responses. |
| Incomplete outcome data addressed (attrition bias) | High risk | Quote: “Participants were family physicians who graduated from the Medical University of South Carolina (MUSC) Family Medicine Residency Program between the years of 1982 and 1999. (and) Of the 182 surveys that were sent, 104 were returned within the 8-week time period.”  Comment: The authors do not report if all family medicine graduates in the study period were mailed surveys. Not all surveys were returned. |
| Selective reporting (reporting bias) | Low risk | Quote: “We hypothesize that Balint training may be positively associated with empathy and work satisfaction.”  Comment: The authors’ hypothesis was that there would be an association between Balint training with empathy and work satisfaction. They reported both in their results. Study protocol available. |

**Journal Article:**

Shapiro J, Morrison E, Boker J. Teaching empathy to first year medical students: evaluation of an elective literature and medicine course. *Educ Health (Abingdon).* 2004;17(1):73-84.

| **Entry** | **Judgement** | **Support for judgement** |
| --- | --- | --- |
| Random sequence generation (selection bias) | Low risk | Quote: “Students who volunteered for the study were randomly assigned to either an immediate intervention group (Group 1: participation in the literature and medicine course) or a wait-list group (Group 2: delayed intervention) by a research assistant using a random number table (Figure 1).” |
| Allocation concealment (selection bias) | High risk | Quote: “All first year students (n = 92) at a public, allopathic medical school in Southern California were invited by e-mail, flier and personal announcement to enroll in a literature and medicine elective and participate in a study of its effects on student attitudes.” (And) “...it is possible that students enrolled in the elective were more empathic than their non-participating classmates.”  Comment: Students motivated to explore and interested in literature and the patient experience enrolled in this elective. So the allocation to participate in either group was biased. |
| Blinding of participants and personnel (performance bias) | High risk | Quote: “All first year students (n = 92) at a public, allopathic medical school in Southern California were invited by e-mail, flier and personal announcement to enroll in a literature and medicine elective and participate in a study of its effects on student attitudes.”  Comment: Student participants were not blinded as to the purpose of the course or the overall study |
| Blinding of outcome assessment (detection bias)  ECRS  BEES | High risk | Quote: “Enroll in a literature and medicine elective and participate in a study of its effects on student attitudes.”  Comment: The announcement of the study to recruit subjects appears to state the outcome assessment. Both were self-assessment and therefore unblinded. |
| Incomplete outcome data addressed (attrition bias) | High risk | Quote: “Sixteen of the 22 volunteer students (73%) completed all pre- and post-quantitative measures. (and) Finally, a problem with missing data reduced the statistical power and forced us to use less powerful nonparametric inferential tests.” |
| Selective reporting (reporting bias) | Unclear risk | Quote: “We tested the hypothesis that reading and discussing literature about patients’ experiences of illness and the doctor–patient relationship could significantly improve medical student empathy and appreciation of the relevance of the humanities for professional development.”  Comment: The study protocol was no available. The authors did pre-specify outcome measures. |
| Other Bias:  (small sample size) | Unclear risk | Quote: “...small number of self-selected subjects and the fact that subjects were recruited from a single institution.” (and) “...unable to administer measures to the whole class, it is possible that students enrolled in the elective were more empathic than their non-participating classmates.”  Comment: Limited generalizability. |

**Journal Article:**

Roter DL, Larson S, Shinitzky H, et al. Use of an innovative video feedback technique to enhance communication skills training. *Med Educ.* 2004;38(2):145-157.

| **Entry** | **Judgement** | **Support for judgement** |
| --- | --- | --- |
| Random sequence generation (selection bias) | High risk | Quote: “Participants were 28 paediatric residents in their first year of postgraduate medical training in a large urban medical centre.”  Comment: This was a pre and post evaluation of a type communication skills training. There was no randomization of participants. The authors did not a describe how these participants were selected. |
| Allocation concealment (selection bias) | High risk | Comment: Participants could forsee assignment as all participants took part in the pre and post evaluation as well as the training program |
| Blinding of participants and personnel (performance bias) | High risk | Comment: Participants were not blinded. The authors do not describe the enrollment process. |
| Blinding of outcome assessment (detection bias)  Roter Interaction Analysis | Low risk | Quote: “Each coder was assigned only one interview per resident and coders were blind to the pre or post feedback status of the interview. (and) A second video-based study using the digital software platform was completed.”  Comment: Coding of video interviews were blinded or completed by a software program |
| Incomplete outcome data addressed (attrition bias) | Low risk | Comment: The authors do not comment specifically whether or not any data is missing but it appears from the results tables that all 28 participants completed the study and both pre and post training video-taped interviews |
| Selective reporting (reporting bias) | Unclear risk | Quote: “There were three study objectives: 1) to explore the acceptability of an innovative video feedback programme to residents and faculty; 2) to evaluate a brief teaching intervention comprising the video feedback innovation when linked to a one-hour didactic and role-play teaching session on paediatric residents’ communication with a simulated patient; and 3) to explore the impact of resident gender on communication change.”  Comment: The authors state their objectives and then report on each in the results. However, study protocol not available. |
| Other Bias:  (small sample size) | Unclear risk | Comment: The automated analysis system has never been validated, small sample size, the authors mention that faculty members preferred this method of feedback to traditional but there were only four faculty members participating. |

**Journal Article:**

Winefield HR, Chur-Hansen A. Evaluating the outcome of communication skill teaching for entry-level medical students: does knowledge of empathy increase? *Med Educ.* 2000;34(2):90-94.

| **Entry** | **Judgement** | **Support for judgement** |
| --- | --- | --- |
| Random sequence generation (selection bias) | High risk | Quote: “Participants included most of a class of 115 first-year medical students (n  107, 93%).”  Comment: The authors do not report any randomization of participants. |
| Allocation concealment (selection bias) | High risk | Quote: “Participants included most of a class of 115 first-year medical students (n 107, 93%).”  Comment: Participants all took the communication training course. There was no control arm of students. |
| Blinding of participants and personnel (performance bias) | High risk | Quote: “In order that we may assess the effectiveness of this Practical in teaching basic medical communication skills, students are asked to complete the task below. Your participation is voluntary, anonymous, and designed to allow evaluation of this project.”  Comment: Students knew they were being tested on improvement of communication skills. |
| Blinding of outcome assessment (detection bias)  Written empathy test | High risk | Quote: “In order that we may assess the effectiveness of this practical in teaching basic medical communication skills, students are asked to complete the task below. Your participation is voluntary, anonymous, and designed to allow evaluation of this project.”  Comment: Students knew they were being tested on improvement of communication skills. |
| Incomplete outcome data addressed (attrition bias) | High risk | Quote: “Table 2 shows the empathy total scores before and after training, for students of each sex whose responses could be matched up.”  Comment: 107 participated and only 96 participants pre and post communication training tests could be matched. The authors do not explain the missing data |
| Selective reporting (reporting bias) | Unclear risk | Quote: “By describing our teaching methods and their outcomes, as measured with a reliable written instrument, we aim to share experiences relevant to facilitating improved communication practice in health care.” (and) “The outcome of training was assessed using a 10-item empathy scale (see Table 1) which requires respondents to write brief responses to trigger statements. The difference between the two total empathy scores over the 10 items showed the degree of either improvement or, where the baseline score was higher than the post-test, deterioration in skills.”  Comment: The authors do not clearly state in advance what their primary outcome is; they merely state they want to share their experience with a communication training program. In the methods they describe an empathy scale and then later in the methods describe using the scale as a before and after measurement. Study protocol was not available. |
| Other Bias:  (other) | High risk | Comment: Results of pre- and post testing are attributed to the course, but there was no control arm of students matriculating through the semester without this course. |

**Journal Article:**

Moorhead R, Winefield H. Teaching counselling skills to fourth-year medical students: a dilemma concerning goals. *Fam Pract.* 1991;8(4):343-346.

| **Entry** | **Judgement** | **Support for judgement** |
| --- | --- | --- |
| Random sequence generation (selection bias) | High risk | Quote: “Successive groups of fourth-year students (N=63) completed the Empathy Rating Scale on two occasions:once at the beginning and once at the end of their initial week’s attachment to General Practice Training…”  Comment: There was no randomization. |
| Allocation concealment (selection bias) | High risk | Quote: “Successive groups of fourth-year students (N=63) completed the Empathy Rating Scale on two occasions:once at the beginning and once at the end of their initial week’s attachment to General Practice Training…”  Comment: Allocation was unconcealed. There was no randomization, and no control arm. |
| Blinding of participants and personnel (performance bias) | Unclear risk | Comment: The authors do not comment on the participants knowledge of the study. |
| Blinding of outcome assessment (detection bias)  Empathy rating scale | Unclear risk | Quote: “The students written responses to the 10 trigger statements of the Empathy Rating Scale were rated without knowledge of which were made before, and which were made after, training in communication.”  Comment: The authors do not comment on whether raters knew the students (participants) or whether or not they were coded and anonymous. |
| Incomplete outcome data addressed (attrition bias) | High risk | Quote: “A further 20 students completed the pre-test only and two students returned incomplete forms.”  Comment: A large portion of students did not complete the post-test. |
| Selective reporting (reporting bias) | Unclear risk | Comment: The authors do not provide any pre-specified outcome measures. |
| Other Bias:  (social desirability bias) | High risk | Comment: It is possible that in this small training program where the participant subjects were all students that their answers were biased to please their instructors. |
| Other Bias:  (Hawthorne effect) | Unclear risk | Comment: It is not clear if the students knew they were being studied. The tendency to perform better when participating in an experiment may have altered behavior. |

**Journal Article:**

Kramer D, Ber R, Moore M. Increasing empathy among medical students. *Med Educ.* 1989;23(2):168-173.

| **Entry** | **Judgement** | **Support for judgement** |
| --- | --- | --- |
| Random sequence generation (selection bias) | High risk | Quote: “Students worked on the ward in self-assigned groups of 10. Four consecutive groups were randomly assigned to one of the following conditions: (I) A control group, with no workshop for either students or tutors; (2) Experimental group A, containing students whose tutors had participated in a supporting medical-interview workshop; (3) Experimental group B, in which the students themselves participated in such a workshop; and (4) Experimental group C, in which both students and their tutors participated in the workshop.”  Comment: Students self selected into groups and then the groups were randomized but they do not report how they randomized the group. |
| Allocation concealment (selection bias) | Unclear risk | Comment: No method of concealment is described. |
| Blinding of participants and personnel (performance bias) | High risk | Quote: “Experimental group A, containing students whose tutors had participated in a supporting medical-interview workshop; Observation of student/doctor behaviour during medical interviews was performed by two trained observers.”  Comment: Participants knew that they were receiving a class and then were aware of being observed. |
| Blinding of outcome assessment (detection bias)  Third party observers | Unclear risk | Quote: “Observation of student/doctor behaviour during medical interviews was performed by two trained observers.”  Comment: The authors do not describe blinding of the observers of student doctor behaviors. |
| Incomplete outcome data addressed (attrition bias) | Unclear risk | Quote: “The means of these observed behaviours were the basis for determining ‘net supporting’ behaviour.”  Comment: The study did not address whether or not there was incomplete outcome data; they did not report whether all subjects had observed encounters and completed the study. |
| Selective reporting (reporting bias) | Unclear risk | Comment: The study did not address whether or not there was selective outcome reporting. The study protocol was not available. |
| Other Bias  (Hawthorne effect) | Unclear risk | The tendency to perform better when participating in an experiment. |

**Journal Article:**

Poole AD, Sanson-Fisher RW. Long-term effects of empathy training on the interview skills of medical students. *Patient Couns Health Educ.* 1980;2(3):125-127.

| **Entry** | **Judgement** | **Support for judgement** |
| --- | --- | --- |
| Random sequence generation (selection bias) | Unclear risk | Quote: “The experimental group was randomly selected from those students who had participated in the empathy training program as part of their second-year course in behavioral science.”  Comment: The authors do not describe how the experimental group was randomly selected |
| Allocation concealment (selection bias) | Unclear risk | Comment: The method of concealment is not described |
| Blinding of participants and personnel (performance bias) | High risk | Quote: “Each subject was given the definition of empathy and an outline of the Accurate Empathy Scale, and was asked to demonstrate his or her ability to empathize during a 15-minute interview with a patient.”  Comment: There is no mention of blinding and the subjects were given information on empathy and the scale by which they would be measured. |
| Blinding of outcome assessment (detection bias)  Accurate Empathy Scale | Unclear risk | Quote: “The interview was recorded, and three two-minute segments were selected at random from each tape recording for subsequent assessment by a trained rater using the nine-point Accurate Empathy Scale.”  Comment: The authors do not mention blinding of raters. |
| Incomplete outcome data addressed (attrition bias) | Unclear risk | Comment: In Table II the authors report data for 25 in the experimental group and 20 in the control group which would yield complete data for their study; but by the methodology of this study it is a look back three years after empathy training and it is not clear how many students originally took the class and whether or not many potential subjects were lost to follow up and unable to be randomized for participation. |
| Selective reporting (reporting bias) | Unclear risk | Quote: “The present study reports the results obtained by reassessing a sample of the students three years after they had completed the training.”  Comment: The authors do not clearly state primary outcome nor now it will be measured |
| Other Bias:  (maturation bias) | Unclear risk | Quote: “It should be noted that the level of empathy of the experimental group obtained at the three-year follow-up was still significantly higher than it had been prior to training.”  Comment: There may have been a natural change/improvement over time. |
| Other Bias:  (Hawthorne effect) | High risk | Quote: “Each subject was given the definition of empathy and an outline of the Accurate Empathy Scale, -01 and was asked to demonstrate his or her ability to empathize during a 15-minute interview with a patient.”  Comment: The tendency to perform better when participating in an experiment may have altered behavior. |
| Other Bias:  (small sample size) | Unclear risk | Comment: The small sample size limits generalizability |

**Journal Article:**

Junek W, Burra P, Leichner P. Teaching interviewing skills by encountering patients. *J Med Educ.* 1979;54(5):402-407.

| **Entry** | **Judgement** | **Support for judgement** |
| --- | --- | --- |
| Random sequence generation (selection bias) | High risk | Quote: “All six first-year residents in the Department of Psychiatry program.”  Comment: There was no randomization. |
| Allocation concealment (selection bias) | High risk | Comment: There was no randomization, and no control arm. All of the first year residents in the residency program participated |
| Blinding of participants and personnel (performance bias) | High risk | Quote: “At the first meeting the instructions, goals of the seminar, research aspect, and method of conducting each subsequent session were reviewed.”  Comment: Participants were not blinded |
| Blinding of outcome assessment (detection bias)  Modified Barrett-Lennard Relationship Inventory | High risk | Quote: “Rated by examiners who did not know the residents.” (and) “The raters used a modified Barrett-Lennard Relationship Inventory.” (and) “Each interviewer was rated on four categories…”  Comment: Although the raters did not know the residents, there is no mention of raters being blinded. |
| Incomplete outcome data addressed (attrition bias) | Unclear risk | Quote: “Five residents completed a 40-to-60-minute videotape of an interview with a patient before and after the seminar series. One resident did not complete his videotaped interviews because he felt too insecure to expose himself on videotape”  Comment: One of the six participants in the seminar was not rated. |
| Selective reporting (reporting bias) | Unclear risk | Quote: “Each interviewer was rated on four categories of empathy, congruence, regard and unconditionality.”  Comment: The authors do not provide any pre-specified outcome measures and the study protocol is not available. |
| Other Bias:  (social desirability bias) | Unclear risk | Comment: It is possible that in this small training program where the participant subjects were all residents that their answers were biased to please their supervisors. |
| Other Bias:  (Maturation bias) | High risk | Comment: Residents may have improved their interviewing skills over a three month period even without the seminar. |
| Other Bias:  (Hawthorne effect) | High risk | Comment: The tendency to perform better when participating in an experiment may have lead to higher post test scores. |
| Other Bias:  (small sample size) | High risk | Comment: Small sample size limits generalizability |

**Journal Article:**

Sanson-Fisher RW, Poole AD. Training medical students to empathize: an experimental study. *Med J Aust.* 1978;1(9):473-476.

| **Entry** | **Judgement** | **Support for judgement** |
| --- | --- | --- |
| Random sequence generation (selection bias) | High risk | Quote: “The experimental group was made up of all students who were taking a second-year unit in Behavioural Science.”  Comment: There was no randomization; there were experimental and control groups assigned based on timing of enrollment in a course in Behavioural Science. The intervention was the Tune-In Empathy Training Workshop. |
| Allocation concealment (selection bias) | High risk | Quote: “The experimental group was made up of all students who were taking a second-year unit in Behavioural Science.”  Comment: There was no allocation concealment. Subjects in experimental arm were chosen because they were enrolled in the course. |
| Blinding of participants and personnel (performance bias) | Unclear risk | Quote: “They were also aware that the interaction would be tape-recorded and, subsequently, rated to assess their level of empathy.”  Comment: There is no mention of blinding of participants or whether or not they knew the objective of the study |
| Blinding of outcome assessment (detection bias)  Accurate Empathy Scale | Unclear risk | Quote: “Three two-minute segments were selected at random from each tape-recording for assessment by a trained rater and the level of empathy assigned to each subject was the average of of these three ratings.”  Comment: There is no mention of blinding of the raters. |
| Incomplete outcome data addressed (attrition bias) | Low risk | Quote: “An experimental and control group of 135 preclinical medical students was employed in this study.”  Comment: Data is reported for all 135 subjects. And, there is no mention of incomplete data. |
| Selective reporting (reporting bias) | Unclear risk | Quote: “...employ the Accurate Empathy Scale to assess the training programme used in this study and to centre the training itself on the acquisition of the ability to to empathize.”  Comment: The authors describe their protocol. But the primary objective and how it would be measured are not clearly stated. The authors do not report statistical significance. |
| Other Bias:  (social desirability bias) | High risk | Quote: “All subjects were provided with a general definition of empathy, and a description of the criteria for assessing empathy on the nine-point Accurate Empathy Scale.”  Comment: Participants may have changed behaviour in a way that would be viewed as favorably by others. |
| Other Bias:  (Hawthorne effect) | Unclear risk | Comment: Participants may have known or been able to discern that their empathy improvement was being tested. Tendency to perform better when participating in an experiment may have lead to higher post test scores. |

**Journal Article:**

Fine VK, Therrien ME. Empathy in the doctor-patient relationship: skill training for medical students. *J Med Educ.* 1977;52(9):752-757.

| **Entry** | **Judgement** | **Support for judgement** |
| --- | --- | --- |
| Random sequence generation (selection bias) | High risk | Quote: “Twenty volunteers who chose to have the training during the fall semester comprised the experimental group. Twenty-three volunteers who elected to be trained in the spring semester served as a control group.”  Comment: There was no randomization. Allocation was by students preference to take the training course in the first semester |
| Allocation concealment (selection bias) | High risk | Quote: “Twenty volunteers who chose to have the training during the fall semester comprised the experimental group.”  Comment: Allocation was unconcealed. |
| Blinding of participants and personnel (performance bias) | Unclear risk | Comment: It is unclear whether or not the participants were blinded to the purpose of the study. They would know that the are learning and being tested on empathy, but there is not mention that they gave informed consent to participate. |
| Blinding of outcome assessment (detection bias)  Traux Accurate Empathy Scale | Low risk | Quote: “When each audiotaped problem was presented, students were asked to write the exact words they would say in response. The responses were typed and coded so that raters were blind to pre-post and experimental-control designations.”  Comment: Raters were blinded. |
| Incomplete outcome data addressed (attrition bias) | Unclear risk | Comment: The authors do not comment on whether or not all participants completed all aspects of the study. |
| Selective reporting (reporting bias) | Unclear risk | Quote: “Was designed to test the hypothesis that students who participated in the skill training program would respond to patient messages with empathy. It was also predicted that their responses would reveal their perception of a patient with a medical problem rather than a medical problem alone.”  Comment: The authors do not pre-specify primary outcome measures and study protocol was not available. |
| Other Bias:  (Hawthorne effect) | Unclear risk | Comment: Tendency to perform better when participating in an experiment may have lead to higher post test scores. |
| Other Bias:  (small sample size) | Unclear risk | Comment: Small sample size limits generalizability |
| Other Bias:  (other) | Unclear risk | Quote: “Twenty volunteers who chose to have the training during the fall semester comprised the experimental group.”  Comment: The students who elected to take the course early may have been more motivated to learn and master the course material. |

**Journal Article:**

Pacoe LV, Naar R, Guyett IP, Wells R. Training medical students in interpersonal relationship skills. *J Med Educ.* 1976;51(09):743-750.

| **Entry** | **Judgement** | **Support for judgement** |
| --- | --- | --- |
| Random sequence generation (selection bias) | High risk | Quote: “The subjects for this study were 13 first-year medical students who volunteered to participate in a training course and seven additional students from the same class who agreed to complete several scales as part of a research project.”  Comment: There was no randomization. Allocation was by students preference to take the training course. |
| Allocation concealment (selection bias) | High risk | Quote: “Allocation was by students preference to take the training course.”  Comment: Allocation was unconcealed. |
| Blinding of participants and personnel (performance bias) | Unclear risk | Quote: “13 first-year medical students who volunteered to participate in a training course and seven additional students from the same class who agreed to complete several scales as part of a research project.”  Comment: It is unclear whether or not the participants were blinded to the purpose of the study. Those taking the course knew that the are learning and being tested on empathy, but there is not mention that they gave informed consent to participate. The controls agreed to take part in a research project but there is no mention of blinding. |
| Blinding of outcome assessment (detection bias)  Empathetic Communication test  Facial Discrimination | Low risk | Quote: “Each of the written responses to the 10 videotaped presentations was typed on cards and coded and pooled so that judges would be blind to pre-post or experimental-control designations.”  Comment: Raters were blinded. |
| Incomplete outcome data addressed (attrition bias) | Unclear risk | Comment: It appears from Table 1 that all participants data was incorporated but the authors do not comment on whether all participants completed all portions of the study. |
| Selective reporting (reporting bias) | Unclear risk | Quote: “In order to determine changes in the level of the core qualities, three groups of dependent variables were assessed…”  Comment: The authors do not pre-specify primary outcome measures and study protocol not available. |
| Other Bias:  (Small sample size) | High risk | Comment: Small sample size limits generalizability |
| Other Bias:  (other) | Unclear risk | Quote: “...13 first-year medical students who volunteered to participate in a training course”  Comment: The students who elected to take the course early may have been more motivated to learn and master the course material. |

PGY-1, Post Graduate Year-1; HRQ, The Helpful Responses Questionnaire; MBI-HSS, Maslach Burnout Inventory—Human Services Survey; SP, Standardized Patient; JSPE, Jefferson Scale of Physician Empathy; OCSE, objective clinical structured examination; JSPPPE, Jefferson Scale of Patient Perceptions of Physician Empathy; JSE, Jefferson Scale of Empathy; CARE, Consultation and Relational Empathy; IRI, Interpersonal Reactivity Index Empathy Scale; HEAT, hear, empathize, apologize, take action; VP, virtual patient; SPA, Standardized Patient Assessment; HCAHPS, Hospital Consumer Assessment of Healthcare Providers and Systems; CGCAHPS, Clinician and Group Consumer Assessment of Healthcare Providers and Systems; ED, Emergency Department; MBI, Maslach Burnout Inventory; SCI - spinal cord injury; FM, family medicine; OAE, objective assessment of empathy; SES, socioeconomic status; HCP, Health Care Provider; NECN, North of England Cancer Network; ACST, Advanced Communication Skills Training; GATA, Gulhane Military Medical Academy

| **References**    1. Bentley PG, Kaplan SG, Mokonogho J. Relational Mindfulness for Psychiatry Residents: a Pilot Course in Empathy Development and Burnout Prevention. *Acad Psychiatry.* 2018.  2. Dotters-Katz SK, Chuang A, Weil A, Howell JO. Developing a pilot curriculum to foster humanism among graduate medical trainees. *J Educ Health Promot.* 2018;7:2.  3. Wundrich M, Schwartz C, Feige B, Lemper D, Nissen C, Voderholzer U. Empathy training in medical students - a randomized controlled trial. *Med Teach.* 2017;39(10):1096-1098.  4. Schweller M, Ribeiro DL, Celeri EV, de Carvalho-Filho MA. Nurturing virtues of the medical profession: does it enhance medical students' empathy? *Int J Med Educ.* 2017;8:262-267.  5. LoSasso AA, Lamberton CE, Sammon M, et al. Enhancing Student Empathetic Engagement, History-Taking, and Communication Skills During Electronic Medical Record Use in Patient Care. *Acad Med.* 2017;92(7):1022-1027.  6. Ruiz-Moral R, Perula de Torres L, Monge D, Garcia Leonardo C, Caballero F. Teaching medical students to express empathy by exploring patient emotions and experiences in standardized medical encounters. *Patient Educ Couns.* 2017;100(9):1694-1700.  7. Buffel du Vaure C, Lemogne C, Bunge L, et al. Promoting empathy among medical students: A two-site randomized controlled study. *J Psychosom Res.* 2017;103:102-107.  8. Zazulak J, Sanaee M, Frolic A, et al. The art of medicine: arts-based training in observation and mindfulness for fostering the empathic response in medical residents. *Med Humanit.* 2017;43(3):192-198.  9. Delacruz N, Reed S, Splinter A, et al. Take the HEAT: A pilot study on improving communication with angry families. *Patient Educ Couns.* 2017;100(6):1235-1239.  10. Flint H, Meyer M, Hossain M, Klein M. Discussing Serious News. *American Journal of Hospice & Palliative Medicine.* 2017;34(3):254-257.  11. Ditton-Phare P, Sandhu H, Kelly B, Kissane D, Loughland C. Pilot Evaluation of a Communication Skills Training Program for Psychiatry Residents Using Standardized Patient Assessment. *Acad Psychiatry.* 2016;40(5):768-775.  12. Boissy A, Windover AK, Bokar D, et al. Communication Skills Training for Physicians Improves Patient Satisfaction. *J Gen Intern Med.* 2016;31(7):755-761.  13. Foster A, Chaudhary N, Kim T, et al. Using Virtual Patients to Teach Empathy: A Randomized Controlled Study to Enhance Medical Students' Empathic Communication. *Simul Healthc.* 2016;11(3):181-189.  14. Orloski CJ, Tabakin ER, Myers JS, Shofer FS, Mills AM. Grab a SEAT: Sit, engage, ask, teach an emergency department performance improvement initiative. *Ann Emerg Med.* 2016;68:4 Supplement 1 (S130-)  15. Duke P, Grosseman S, Novack DH, Rosenzweig S. Preserving third year medical students' empathy and enhancing self-reflection using small group "virtual hangout" technology. *Med Teach.* 2015;37(6):566-571.  16. Lusilla-Palacios P, Castellano-Tejedor C. Training a Spinal Cord Injury Rehabilitation Team in Motivational Interviewing. *Rehabil Res Pract.* 2015;2015:358151.  17. Potash JS, Chen JY, Lam CL, Chau VT. Art-making in a family medicine clerkship: how does it affect medical student empathy? *BMC Med Educ.* 2014;14:247.  18. Nasr Esfahani M, Behzadipour M, Jalali Nadoushan A, Shariat SV. A pilot randomized controlled trial on the effectiveness of inclusion of a distant learning component into empathy training. *Med J Islam Repub Iran.* 2014;28:65.  19. Williams B, Sadasivan S, Kadirvelu A, Olaussen A. Empathy levels among first year Malaysian medical students: an observational study. *Adv Med Educ Pract.* 2014;5:149-156.  20. Airagnes G, Consoli SM, De Morlhon O, Galliot AM, Lemogne C, Jaury P. Appropriate training based on Balint groups can improve the empathic abilities of medical students: a preliminary study. *J Psychosom Res.* 2014;76(5):426-429.  21. Schweller M, Costa FO, Antonio MA, Amaral EM, de Carvalho-Filho MA. The impact of simulated medical consultations on the empathy levels of students at one medical school. *Acad Med.* 2014;89(4):632-637.  22. Graham KL, Green S, Kurlan R, Pelosi JS. A patient-led educational program on Tourette Syndrome: impact and implications for patient-centered medical education. *Teach Learn Med.* 2014;26(1):34-39.  23. Bays AM, Engelberg RA, Back AL, et al. Interprofessional communication skills training for serious illness: evaluation of a small-group, simulated patient intervention. *J Palliat Med.* 2014;17(2):159-166.  24. Tang L, Pang Y, Wang Y, He Y, Song L. Preliminary effects of oncology balint workshop in China. *Psycho-Oncology* 2014;23 SUPPL. 3 303-304.  25. Yang KT, Yang JH. A study of the effect of a visual arts-based program on the scores of Jefferson Scale for Physician Empathy. *BMC Med Educ.* 2013;13:142.  26. Gibon AS, Merckaert I, Lienard A, et al. Is it possible to improve radiotherapy team members’ communication skills? A randomized study assessing the efficacy of a 38-h communication skills training program. *Radiother Oncol.*  2013;109(1): 170-7. |
| --- |

27. Johnson LA, Gorman C, Morse R, Firth M, Rushbrooke S. Does communication skills training make a difference to patients' experiences of consultations in oncology and palliative care services? *Eur J Cancer Care (Engl).* 2013;22(2):202-209.

28. Blanco MA, Maderer A, Price LL, Epstein SK, Summergrad P. Efficiency is not enough; you have to prove that you care: role modelling of compassionate care in an innovative resident-as-teacher initiative. *Educ Health (Abingdon).* 2013;26(1):60-65.

29. Riess H, Kelley JM, Bailey RW, Dunn EJ, Phillips M. Empathy training for resident physicians: a randomized controlled trial of a neuroscience-informed curriculum. *J Gen Intern Med.* 2012;27(10):1280-1286.

30. Cinar O, Ak M, Sutcigil L, et al. Communication skills training for emergency medicine residents. *Eur J Emerg Med.* 2012;19(1):9-13.

31. Ozcan CT, Oflaz F, Bakir B. The effect of a structured empathy course on the students of a medical and a nursing school. *Int Nurs Rev.* 2012;59(4):532-538.

32. Lim BT, Moriarty H, Huthwaite M. "Being-in-role": A teaching innovation to enhance empathic communication skills in medical students. *Med Teach.* 2011;33(12):e663-669.

33. Tulsky JA, Arnold RM, Alexander SC, et al. Enhancing communication between oncologists and patients with a computer-based training program: a randomized trial. *Ann Intern Med.* 2011;155(9):593-601.

34. Riess H, Kelley JM, Bailey R, Konowitz PM, Gray ST. Improving empathy and relational skills in otolaryngology residents: a pilot study. *Otolaryngol Head Neck Surg.* 2011;144(1):120-122.

35. Cahan MA, Larkin AC, Starr S, et al. A human factors curriculum for surgical clerkship students. *Arch Surg.* 2010;145(12):1151-1157.

36. Sripada BN, Henry DB, Jobe TH, Winer JA, Schoeny ME, Gibbons RD. A randomized controlled trial of a feedback method for improving empathic accuracy in psychotherapy. Psychol Psychother. 2011;84(2):113-127.

37. Ghetti C, Chang J, Gosman G. Burnout, psychological skills, and empathy: balint training in obstetrics and gynecology residents. *J Grad Med Educ.* 2009;1(2):231-235.

38. Bonvicini KA, Perlin MJ, Bylund CL, Carroll G, Rouse RA, Goldstein MG. Impact of communication training on physician expression of empathy in patient encounters. *Patient Educ Couns.* 2009;75(1):3-10.

39. Shapiro SM, Lancee WJ, Richards-Bentley CM. Evaluation of a communication skills program for first-year medical students at the University of Toronto. *BMC Med Educ.* 2009;9:11.

40. Fernndez-Olano C, Montoya-Fernndez J, Salinas-Snchez AS. Impact of clinical interview training on the empathy level of medical students and medical residents. *Med Teach.* 2008;30(3):322-324.

41. Dow AW, Leong D, Anderson A, Wenzel RP, Team VCUT-M. Using theater to teach clinical empathy: a pilot study. *J Gen Intern Med.* 2007;22(8):1114-1118.

42. Cataldo KP, Peeden K, Geesey ME, Dickerson L. Association between Balint training and physician empathy and work satisfaction. *Fam Med.* 2005;37(5):328-331.

43. Shapiro J, Morrison E, Boker J. Teaching empathy to first year medical students: evaluation of an elective literature and medicine course. *Educ Health (Abingdon).* 2004;17(1):73-84.

44. Roter DL, Larson S, Shinitzky H, et al. Use of an innovative video feedback technique to enhance communication skills training. *Med Educ.* 2004;38(2):145-157.

45. Winefield HR, Chur-Hansen A. Evaluating the outcome of communication skill teaching for entry-level medical students: does knowledge of empathy increase? *Med Educ.* 2000;34(2):90-94.

46. Moorhead R, Winefield H. Teaching counselling skills to fourth-year medical students: a dilemma concerning goals. *Fam Pract.* 1991;8(4):343-346.

47. Kramer D, Ber R, Moore M. Increasing empathy among medical students. *Med Educ.* 1989;23(2):168-173.

48. Poole AD, Sanson-Fisher RW. Long-term effects of empathy training on the interview skills of medical students. *Patient Couns Health Educ.* 1980;2(3):125-127.

49. Junek W, Burra P, Leichner P. Teaching interviewing skills by encountering patients. *J Med Educ.* 1979;54(5):402-407.

50. Sanson-Fisher RW, Poole AD. Training medical students to empathize: an experimental study. *Med J Aust.* 1978;1(9):473-476.

51. Fine VK, Therrien ME. Empathy in the doctor-patient relationship: skill training for medical students. *J Med Educ.* 1977;52(9):752-757.

52. Pacoe LV, Naar R, Guyett IP, Wells R. Training medical students in interpersonal relationship skills. *J Med Educ.* 1976;51(09):743-750.
